# Immunotherapies for Aging and Age-Related Diseases: Advances, Pitfalls, and Prospects

**DOI:** 10.34133/research.0866

**Published:** 2025-09-08

**Authors:** Muyang Yang, Shipeng Wu, Jiasi Zhang, Lisen Lu, Deqiang Deng, Qianfeng Xia, Jonathan F. Lovell, Xiujuan Shi, Honglin Jin

**Affiliations:** ^1^ NHC Key Laboratory of Tropical Disease Control, School of Life Sciences and Medical Technology, Hainan Medical University, Haikou, Hainan 571199, China.; ^2^ College of Biomedicine and Health and College of Life Science and Technology, Huazhong Agricultural University, Wuhan, Hubei 430070, China.; ^3^ Institution of Pediatric Hematology & Oncology, Tongji Hospital, Tongji Medical College, Huazhong University of Science and Technology, Wuhan, Hubei 430030, China.; ^4^ Department of Biomedical Engineering, University at Buffalo, State University of New York, Buffalo, NY 14260, USA.

## Abstract

Aging is characterized by a gradual decline in the functionality of all the organs and tissues, leading to various diseases. As the global population ages, the urgency to develop effective anti-aging strategies becomes increasingly critical due to the growing severity of associated health problems. Immunotherapy offers novel and promising approaches to combat aging by utilizing approaches including vaccines, antibodies, and cytokines to target specific aging-related molecules and pathways. In this review, we summarize the recent advancements of immunotherapies to eliminate senescence cells and senescence-associated secretory phenotypes (SASPs). Next, we describe immunotherapies against age-related diseases containing neurodegenerative diseases, vascular pathologies, type 2 diabetes (T2D), arthritis, osteoporosis, chronic obstructive pulmonary disease (COPD), fibrosis, skin aging, and cancer, detailing the targets, corresponding manipulations, and clinical trials. Related problems including immunosenescence, serious adverse effects, the duality of function of senescent cells and SASPs, and inherent problems of immunotherapy are also discussed, suggesting directions for improvement. In addition to recent development and challenges in the field, we describe a blueprint of personalized aging immunotherapy by multi-omics, Big Data, artificial intelligence (AI), and nanobots in the future, aiming to inspire the development of novel strategies for healthy aging.

## Introduction

Aging begins with the gradual accumulation of “primary damage” to the genome, shortening of telomeres, shifting epigenetics, declining protein homeostasis, and mitochondria disfunction [1]. These insults elicit cellular senescence—an antagonistic response that, when immune surveillance fails with age, sustains senescence-associated secretory phenotype (SASP)-mediated chronic low-grade inflammation, amplifies damage, promotes fibrosis, and ultimately manifests as age-related diseases [2,3]. The global elderly population is projected to rise, with those aged 65 and above reaching nearly 20% by 2050 [4]. Advancing age is one of the most critical factors elevating the risk of numerous chronic and life-threatening diseases. Moreover, a weakened immune system contributes to the prevalence of many endogenous diseases while making the elderly more vulnerable to infections. Thus, it is urgent to explore the mechanisms of aging and develop anti-aging strategies.

Intervention measures for aging and age-related diseases have attracted much attention due to their potential for healthcare and economic benefits. Exercise and calorie restriction not only slow aging itself but also markedly lower the incidence and delay the onset of many age-related diseases [5]. It is an endogenous behavioral intervention, whereas the present review focuses on exogenous therapeutic approaches, such as drugs and immunotherapies. As the fundamental mechanisms of cellular and organ senescence have been revealed, numerous small-molecule drugs and gene therapies have been developed with proven efficacy in anti-aging strategies. Senolytics provide rapid and direct depletion of senescent cell burden, yet they are constrained by hematologic toxicities, off-target effects, and variable efficacy across senescent cell subtypes. Senomorphics/senostatics blunt SASP secretion without eliminating the cells; however, their long-term utility is limited by a lack of specificity, requirement for continuous dosing, rebound SASP upon withdrawal, and the potential to chronically dampen inflammatory pathways that underpin immune surveillance and normal tissue repair [6]. Gene therapies can induce off-target effects and elevate the risk of carcinogenesis due to unpredictable gene insertion [7]. Therefore, there is an urgent need to develop innovative approaches to combat aging and related diseases with minimized side effects.

Immunotherapy, after a century of development, has revolutionized the treatment of many diseases such as infections, cancers, and autoimmune diseases. Multidisciplinary advancements in immunology, molecular biology, biomedical engineering, and computer science have made immunotherapy more precise and specific, expanding its indications. A recent study on mice demonstrated that immune checkpoint blockade (ICB) can enhance senescence surveillance, indicating the potential of immunotherapies for addressing aging [8]. Senescent cells and aging-related microenvironmental features, such as DNA damage, oncogene expression, mitochondrial metabolic changes, and the production of SASPs, provide targets for immunotherapy to eliminate and reshape the microenvironment [9]. Furthermore, immunotherapies can also reverse immunosenescence, which manifests as chronic low-grade inflammation and diminished reactivity toward pathogens and malignancies [10]. These features highlight the potential of immunotherapy in delaying aging and related diseases with fewer side effects.

This review summarizes the latest progress in aging immunotherapy, including vaccines, adoptive cell transfer (ACT), antibody blockade, and cytokine intervention, while discussing the toxicity issues and limitations encountered in practice. Finally, it describes the prospects of aging immunotherapy, providing new targets and strategies for addressing aging and extending lifespan.

## Immunotherapies Targeting Aging Itself

**Fig. 1.**
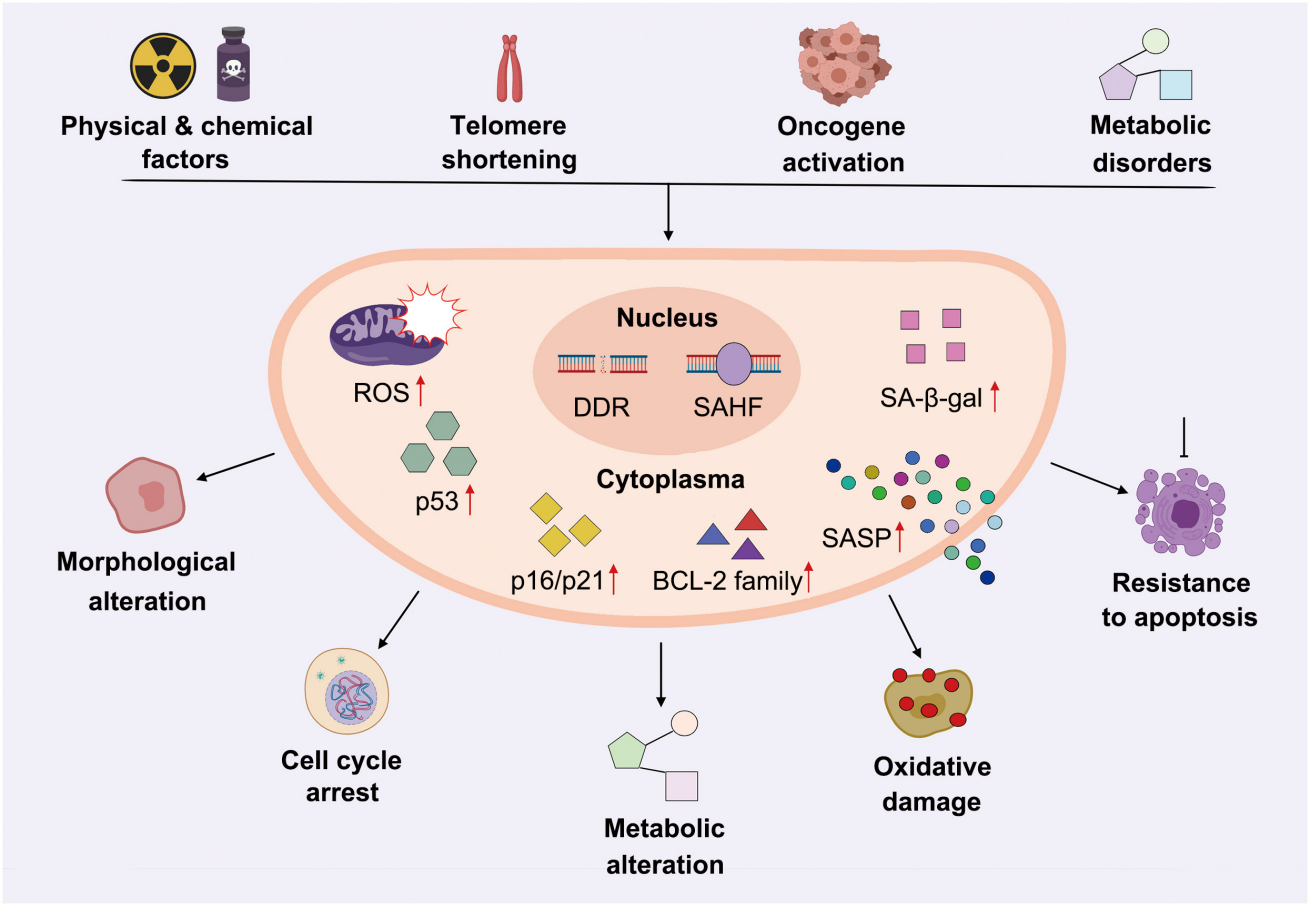
Mechanisms of cellular senescence. Physical and chemical damage, telomere shortening, and oncogene activation lead to DNA damage, triggering the DNA damage response (DDR) and senescence-associated heterochromatin foci (SAHF). This response may promote senescence through elevated levels of ROS, activation of p53, up-regulation of p16, p21, and BCL-2 family, production of SASP, and accumulation of senescence-associated β-gal (SA-β-gal). These cellular changes result in morphological alterations, cell cycle arrest, metabolic shifts, oxidative damage, and increased resistance to apoptosis. The figure is created with MedPeer (medpeer.cn).

Cellular senescence is an irreversible process affecting various pathways, leading to morphological, cyclical, metabolic, and oxidative damage changes (Fig. 1). Among the multiple contributing factors identified to date, the most important include physical and chemical damage, telomere shortening, oncogene activation, and metabolic disorders that activate p53 and up-regulate p16/p21 as well as anti-apoptotic proteins of the B cell lymphoma-2 (BCL-2) family [9]. Aging-related pathways lead to mitochondrial and lysosomal dysfunction, impairing metabolism and promoting reactive oxygen species (ROS) production, thus accelerating senescence [6]. Senescent cells can transmit aging signals to normal cells via abnormal metabolites, microRNAs, SASPs, membrane-bound bodies, and nonvesicular multi-component macromolecules [11]. These alterations result in cellular functional decline and the increase of senescence markers including p16, p21, β-galactosidase (β-gal), and SASPs.

**Fig. 2.**
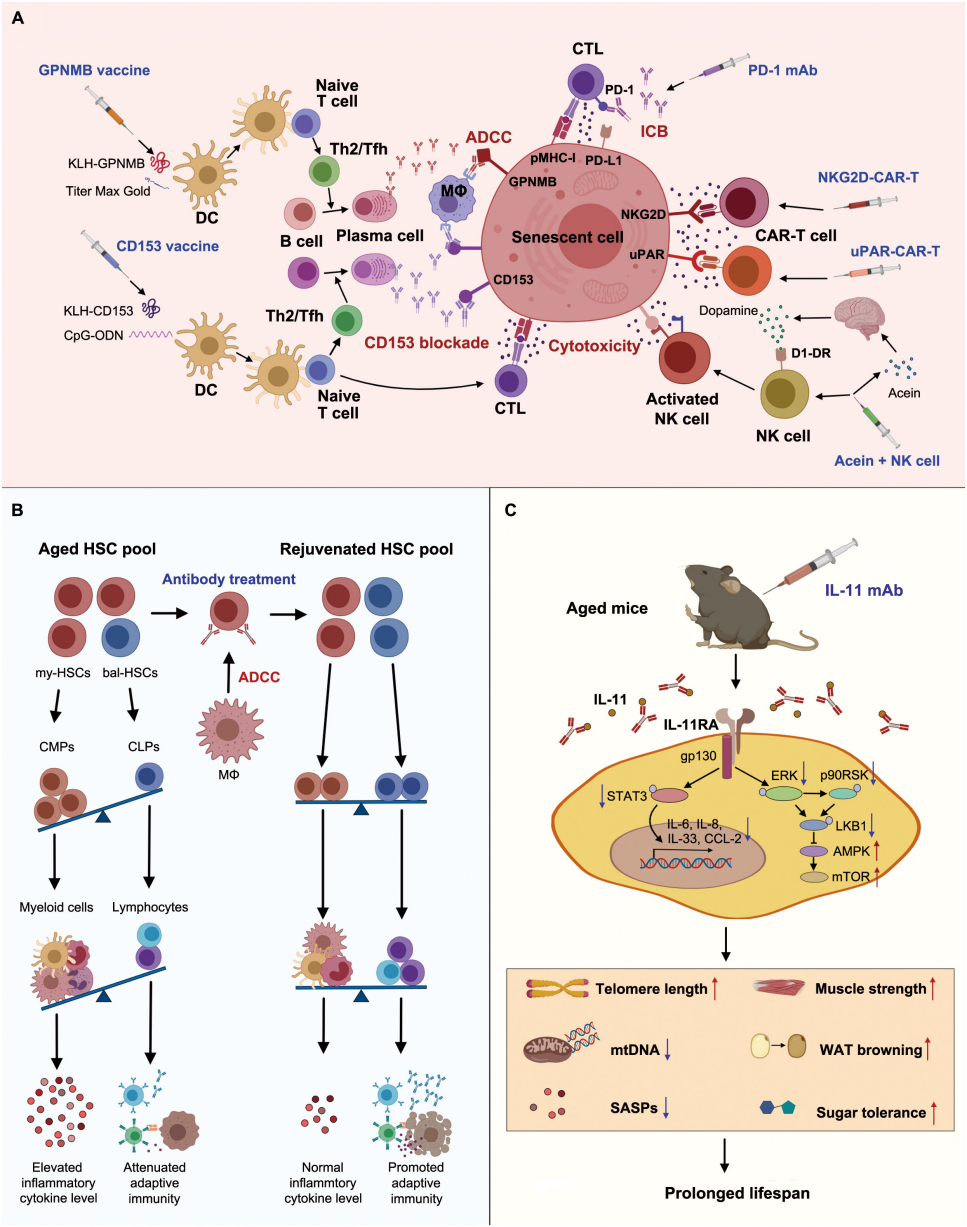
Innovative strategies against aging. (A) Novel approaches to eradicate senescent cells through vaccines, ICB, and ACT. (B) Depletion my-HSCs to rebalance the generation of myeloid cells and lymphocytes for reshaping senescence microenvironment. (C) Ablation of the key SASP IL-11 to prolong the lifespan of old individual. The figure is created with MedPeer (medpeer.cn).

Studies indicate that eliminating senescent cells and their SASPs can restore tissue vitality, prolong lifespan, and delay the onset of many age-related diseases [12]. To reduce nontargeted side effects, identifying aging-related molecules highly expressed in senescent individuals and low in nonsenescent ones is crucial. Recently, new senescence markers have been discovered and used as immunotherapy targets, clearing senescent cells, hematopoietic stem cells (HSCs), and senescence-associated cytokines through vaccines, antibodies, and ACTs [13–19] (Fig. 2). Despite some reports of adverse reactions, the significant effects on aging and innovative designs offer new strategies for healthy aging.

### Senescent cell clearance by vaccines

Strategies for rejuvenating the immune system through the clearance of senescent T cells are crucial for combating aging, thus requiring specific targets on these cells. CD153/CD30 signaling in senescent T cells can accelerate the expansion of tertiary lymphoid structures in aged mice, leading to kidney damage and the development of systemic lupus erythematosus (SLE) [20]. To inhibit the signaling, Yoshida et al. [14] developed a CD153 vaccine composed of CD153 peptide–keyhole limpet hemocyanin (KLH) and the adjuvants [aluminum or CpG-oligodeoxynucleotide (ODN)]. The vaccine containing CpG-ODN can induce anti-CD153 antibodies, clear CD153^+^ senescent T cells, and improve insulin resistance and glucose tolerance in high-fat diet (HFD)-induced obese mice. The effectiveness of CpG-ODN instead of aluminum indicated the importance of killing CD153^+^ senescent T cells by T helper 1 (Th1) response and cellular immunity (Fig. 2A). Additionally, immunoglobulin G2 (IgG2) antibody induced by vaccination can mediate complement-dependent cytotoxicity of senescent T cells. This study demonstrated the feasibility of an anti-aging vaccine targeting CD153. Nevertheless, the vaccine system may promote the progression of autoimmune responses via activation of Th1 cell and CD8^+^ T cells. Moreover, the interaction between CD30 and CD153, as well as the *cis* interaction between CD153 and T cell receptor (TCR)/CD3 complex, is crucial for triggering inflammation against pathogens such as *Mycobacterium tuberculosis* [21]. Blocking CD153 may increase the susceptibility of the elderly to tuberculosis infection. Further research is needed to balance the effective clearance of senescent T cells with the minimization of impacts on autoimmunity and anti-infection to determine the optimal dose, drug compatibility, and administration timing for individual aging and immune status.

In addition to CD153, glycoprotein nonmetastatic melanoma protein B (GPNMB) is another crucial factor associated with aging and age-related diseases. As an endogenous type-1 transmembrane glycoprotein, GPNMB is highly expressed in macrophages and microglia [22]. In 2021, Suda et al. [13] identified GPNMB as a potential candidate for aging-related antigens by transcriptome data from the elderly. In the Gpnmb-diphtheria toxin receptor (DTR) HFD mouse model, metabolic abnormalities and atherosclerosis were alleviated in the absence of GPNMB^+^ cells. Then, a GPNMB vaccine containing KLH-conjugated peptides from GPNMB and Titer Max Gold (adjuvant) was administrated to HFD mice, showing a down-regulation of β-gal, cyclin-dependent kinase inhibitor 1A (CDKN1A), and CDKN2A in visceral adipose tissue. The GPNMB vaccine induced high level of GPNMB antibody, eliminating GPNMB^+^ cells through antibody-dependent cell-mediated cytotoxicity (ADCC), thereby alleviating tissue senescence and age-related phenotypes, and extending the lifespan of mice (Fig. 2A). Similar to CD153, GPNMB also plays a vital role in physiologic function such as immune response initiation, lysosomal integrity, and vascular endothelial cell (VEC) replication [22,23]. Blocking GPNMB may impair vascular endothelial repair, leading to internal hemorrhage. The mechanism of GPNMB in various physiologic processes needs to be thoroughly elucidated, which serves as the foundation to optimize GPNMB vaccination schedule for minimum side effects.

### Senescent cell clearance by elevating T cell cytotoxicity through ICB

Immune checkpoints provide co-inhibitory signals to prevent excessive activation of the immune response and autoimmunity [24]. Programmed cell death protein 1 (PD-1)/programmed cell death 1 ligand 1 (PD-L1) blockade has been successful in cancer treatment and age-related diseases. Onorati et al. [25] found that senescent cells highly express PD-L1 and up-regulate its expression in nonsenescent cells via the activation of the Janus kinase (JAK)–signal transducer and activator of transcription (STAT) pathway, unveiling the relationship between PD-L1 and senescence. Additionally, PD-L1^+^ senescent cells can resist T cell-mediated clearance and cause senescence-associated inflammation by producing high levels of SASPs [8]. PD-L1 plays a key role in the aging process mainly because its expression is affected by factors such as SASPs, low early 2 factor (E2F) activity, and impaired proteasome activity caused by aging [8]. Meanwhile, the senescence marker p16 also participates in maintaining the stability of PD-L1 by weakening its ubiquitin-dependent degradation mediated by cell cycle kinase CDK4/6 [26]. Wang et al. [8] treated p16-creERT2-tdTomato transgenic mice with anti-PD-1 antibodies, reducing p16^+^ cell numbers in the lung, liver, and kidney and improving age-related physiological aging markers. Thus, anti-PD-1 treatment can reduce the inhibitory effects of PD-L1^+^ senescent cells on T cells, achieving normal immune surveillance, senescent cell (SC) elimination, and improvement of age-related phenotype (Fig. 2A). Although anti-PD-1 antibodies are effective in addressing aging, further research is needed to determine the optimal dose and treatment duration to prevent immune-related adverse events (irAEs).

### Senescent cell clearance based on ACTs

Under physiological conditions, the immune surveillance system controls the number of senescent cells via the cytotoxicity of lymphocytes and the phagocytosis of macrophages and granulocytes [27]. Among them, natural killer (NK) cells play a key role in mediating senescent cell clearance through NK group 2 member D (NKG2D)–ligand interaction [28]. However, with immune aging, the numbers, subpopulations, secretory profiles, and functions of NK cells change, leading to a reduction in senescent cell clearance. This has prompted research on ACTs of NK cells against aging. In a small pilot study of five cancer patients, Chelyapov et al. [29] conducted infusions of autologous propagated and activated NK cells into patients, significantly reducing the number of senescent cells in peripheral blood mononuclear cells (PBMCs) and reshaping the cytokine profile while also lowering the levels of SASPs and the number of senescent T cells. The senescent environment, characterized by hormones, cytokines, chemokines, pro-aging cell subpopulations, etc., may impair the efficiency of SC clearance and potentially reshape the phenotypes and function of transferred NK cells. Bai et al. [17] developed an ACT by dopamine-induced activation of autologous NK cells with enhanced NK cell cytotoxicity in aged mice (Fig. 2A). Dopamine not only is crucial for neuroimmune communication but also plays an important role in immune regulation [30]. NK activation can be mediated by interactions between D1-like dopamine receptors (D1-DRs) and dopamine, a process that is hindered in aging due to decreased dopamine levels [31]. According to the fact, a nonapeptide Acein was applied to assist autologous NK cell adoptive transfer via specifically interacting with angiotensin-converting enzyme I (ACE I) to trigger dopamine production without significant neurotoxicity. The results showed that after NK cell ACT and Acein treatment, the levels of p16, p21, and SASPs in multiple tissues decreased, and the nicotinamide adenine dinucleotide (NAD) level improved, supporting the potential of NK cell infusion in anti-aging.

Next to NK cells, cytotoxic T lymphocytes (CTLs) with genetic engineering like chimeric antigen receptor (CAR)-T can also be manipulated to clean senescent cells with special markers. In senescent cells, the high expression of NKG2D ligands (NKG2DLs) not only mediates senescent cell depletion by NK cell-mediated cytotoxicity but also provides a potential target for CAR-T therapy. Yang et al. [32] designed the extracellular domain of NKG2D as CAR (NKG2D-CAR) for recognition of NKG2DLs (Fig. 2A). Animal experiments demonstrated that NKG2D–CAR-T cell administration significantly slowed down senescence progression in aging mice and nonhuman primates as evidenced by reduction of aging markers, alleviation of fat loss, osteoporosis and movement disorders, and improvement of hepatic and renal function without observable systemic toxicity. Another team identified urokinase-type plasminogen activator receptor (uPAR) as a new target for CAR to clear senescent cells [18] (Fig. 2A). The modified CAR-T cells effectively cleared uPAR^+^ senescent cells, improved liver function in liver fibrosis mouse models, reduced the level of SASPs in peripheral blood, and prolonged the lifespan of mice. By single-cell analysis of cells from human liver, fat, and pancreas, the numbers of uPAR^+^ senescent cells were demonstrated to increase with age, indicating the feasibility and potential of uPAR-CAR-T therapy in clinical application [33]. However, transient elevations in liver enzymes were observed in nonhuman primates treated with CAR-T cells, likely attributable to on-target/off-tumor effects [34]. Administration of interleukin-4 (IL-4) restored CAR-T antitumor potency while mitigating hepatotoxicity [35].

### Aging intervention targeting myeloid-biased HSCs

With age, immune function declines, which is related to immunosenescence, and senescent immune cells produce SASPs, mediating persistent low-level inflammation and inducing senescence in other cells. The clearance of senescent immune cells has attracted interest, but their continuous generation after clearance highlights the importance of understanding the formation and development mechanisms of senescent immune cells. HSCs, the progenitors of most immune cells, can regulate the differentiation and proportion of various immune cell types. The role of HSCs has stimulated the scientific community to explore the relationship between HSCs and aging to better understand the mechanisms of immunosenescence and design new intervention measures. In 2005, Rossi et al. [36] discovered differences in HSCs between young and old mice, with myeloid-biased HSCs (my-HSCs) dominating the HSC pool in old mice and humans, leading to reduction in lymphoid-to-myeloid output weakened lymphocyte production and enhanced myeloid cell production. The higher frequency and faster accumulation of mutations associated with myeloid hematopoiesis, compared to those related to lymphoid hematopoiesis, may partly explain my-HSCs bias [37]. Wang et al. [38] discovered that tumor necrosis factor-α (TNF-α) activates extracellular signal–regulated kinase (ERK)–erythroblast transformation specific proto-oncogene 1 (ETS1) signaling pathway in HSCs of old mice, resulting in high expression of IL-27Ra, and thereby skewing these IL-27Ra^+^ HSCs toward myeloid lineage. The finding enlightened the strategies against aging by screening targets for elimination of age-related HSCs. In 2024, Ross et al. [39] identified CD150, CD62p, and Neogenin-1 as their markers across independent datasets of gene expression. Animal experiments showed that the administration of CD150, CD62p, and Neogenin-1 antibodies effectively depleted my-HSCs by ADCC, rebalancing the numbers of common lymphoid progenitors and common myeloid progenitors (Fig. 2B). The aging phenotypes and inflammatory markers were also down-regulated. Antibody treatment significantly increased the number of circulating naive T cells and mature B cells, rejuvenating the adaptive immunity of aged mice to resist infection. However, increased lymphocyte production in the elderly theoretically may promote the incidence of lymphoid cancers, and antibody-mediated my-HSC clearance may damage tissues or physiological functions. Therefore, precise targeting of my-HSCs to avoid toxicity is necessary, and synNotch-CAR technology can be utilized to avoid “on-target/off-cell-type” toxicity [40].

### Aging intervention by cytokine blockade

Disruption of the cytokine profile is a key feature of aging, resulting from the generation of senescent cells and inflammaging. Inflammatory cytokines such as IL-6, IL-1, and TNF-α can trigger persistent inflammation, leading to tissue and organ damage, fibrosis, and aging [41]. Meanwhile, transforming growth factor-β (TGF-β) is involved in immune regulation and is also associated with DNA damage, oxidative stress, and fibrosis [42]. Blocking these cytokines using antibodies, corresponding free receptors, or small-molecule inhibitors is considered to be a potential strategy to alleviate age-related diseases.

IL-11, a member of the IL-6 cytokine family, has recently been identified as a novel target for inhibiting the progression of aging [19]. Early research indicated that IL-11 promotes the development of megakaryocyte colonies and T cell-dependent specific B cells [43]. Its important role in autoimmune diseases and fibrosis was later unveiled [44]. In 2024, Widjaja et al. [19] found that IL-11 levels increase with age, suggesting that IL-11 can serve as a biomarker of aging. By regulating the ERK–adenosine 5′-monophosphate-activated kinase (AMPK)–mammalian target of rapamycin complex 1 (mTORC1) axis, IL-11 accelerates aging pathologies at the cellular, tissue, and organismal levels, manifesting as increased liver and abdominal fat accumulation and decreased muscle mass and strength. In a mouse model, the administration of anti-IL-11 inhibited inflammation in visceral white adipose tissue, expanding the lifespan of female mice by 25% and of male mice by 22.5% (Fig. 2C). Mechanism studies showed that IL-11 blockade can reduce the activity of multiple pathways related to aging and pathways involved in cardiovascular disease, muscle strength, lipid metabolism, telomere attrition, and mitochondrial damage, indicating that it can be a promising therapeutic strategy for aging [19].

## Immunotherapies for Age-Related Diseases

Aging can lead to the onset of various diseases, such as neurodegenerative diseases, vascular pathologies, type 2 diabetes (T2D), arthritis, osteoporosis, chronic obstructive pulmonary disease (COPD), fibrosis, skin aging, and cancer. Advances in immunotherapies have shown promise in ameliorating age-related diseases, potentially contributing to healthy aging. This section focuses on the mechanisms and applications of immunotherapies for age-related diseases.

### Neurodegenerative diseases

Neurodegenerative diseases are characterized by the progressive loss and functional decline of neurons within the central nervous system. These include Alzheimer’s disease (AD), Parkinson’s disease (PD), Lewy body dementia, Huntington’s disease, amyotrophic lateral sclerosis, and progressive supranuclear palsy. Many immunotherapies have been developed for AD and PD in clinical research, which will be described in the following sections (Table 1).

**Table 1.** Summary of clinical trials of the immunotherapies for AD and PD

Name	Target	Indication	Modality	Results of clinical trials
AN1792	Aβ_42_	Mild to moderate AD	Vaccine	The phase II study (NCT00021723) showed that AN1792 induced the sustained production of Aβ antibody and slowed the impairment of cognition. Related postmortem study demonstrated that patients immunized with AN1792 remained virtually plaque-free for 14 years. However, meningoencephalitis occurred in 6% AD patients treated with AN1792, possibly due to the QS21 that activates the autoreactive T cells and microglia. The clinical trial terminated in 2003.
ACI-24	Aβ_42_	Patients with DS	Vaccine	According to the result of phase I study NCT02738450, there was no case of serious adverse events like meningoencephalitis that occurred after ACI-24 vaccine administration among adults with DS. The titers of anti-Aβ antibody were not collated with any adverse findings. However, the following phase II trial has been withdrawn.
UB-311	Aβ_42_	Mild AD	Vaccine	The phase IIa study (NCT02551809) showed that 97% patients have antibody response and 93% of them maintained at 93% by the end of the study. During the treatment, no significant AE occurred except injection site swelling and agitation in 21% patients. According to its effectiveness and safety, UB-311 received FTD from the FDA in May 2022.
CAD106	Aβ_42_	Mild to moderate AD	Vaccine	The results of clinical trials showed that CAD106 has a favorable safety and well tolerability, and the antibody response correlated negatively with Aβ accumulation (NCT00733863, NCT00411580, NCT00795418, NCT00956410, NCT01023685). A recent clinical trial (NCT02565511, phase II/III) combined BACE-1 inhibitor umibecestat and CAD106 was tested in 60- to 75-year-old participants with high genetic risk for AD. Most of participants in the treatment group observed plaque formation deceleration. However, the study was prematurely terminated.
ACC-001	Aβ_42_	Mild to moderate AD	Vaccine	Sustained Aβ IgG antibody responses were observed in patients with mild to moderate AD after ACC-001 administration (NCT00752232, phase II). The vaccine was verified as safe and well-tolerated (NCT01284387, phase II); however, related studies had halted owing to no significant differences of cognitive improvement.
AFFITOPE AD02	Aβ_42_	Prodromal and mild AD	Vaccine	No significant difference of cognitive improvement was observed in clinical trial (NCT01117818, phase II). The following phase II trial (NCT02008513) was terminated.
Lu AF20513	Aβ_42_	Probable AD	Vaccine	The effects of Lu AF20513 did not meet expectations, and the clinical trial (NCT03819699, phase II) was terminated in November 2019.
ALZ-101	Soluble Aβ oligomers	Mild to moderate AD	Vaccine	A phase Ib study (NCT05328115) was completed in January 2025, and the result has not been posted.
AV-1959D	Aβ_42_	Mild AD	Vaccine	A phase I study (NCT05642429) is ongoing.
ABvac 40	Aβ_40_	Mild to moderate AD	Vaccine	The phase I trial of ABvac40 (NCT03113812) reported that the vaccine can boost the specific immune response against C terminus of Aβ with well tolerance and safety. Further study (NCT03461276, phase II) indicates that the therapy is promising due to significant improvement in cognition according to the results of MMSE.
AADvac1	Tau	Mild to moderate AD	Vaccine	Elevated antibody titers, mitigation of atrophy, and improvement of cognition occurred in patients after AADvac1 vaccination (NCT02031198). The phase II study (NCT02579252) showed significant reduction of the levels of 2 CSF biomarkers of AD, including p-tau181 and p-tau217.
ACI-35	Tau	Mild to moderate AD	Vaccine	The phase I/II clinical trial of ACI-35 has been terminated (NCT04445831).
GV1001	hTERT	Mild to moderate AD	Vaccine	The clinical trial (NCT03184467, phase II) showed that GV1001 had greater language benefits than the patients in control group with well tolerance and favorable safety. Further clinical study of GV1001 (NCT05189210) is ongoing.
UB-312	α-Syn	PD	Vaccine	According to the result of the phase I study NCT04075318, the administration of UB-312 did not lead statistical differences of clinical scales for PD; however, it can reduce αSyn seeds in CSF.
Aducanumab	Aβ_42_	Prodromal and mild AD	Antibody	In the phase Ib randomized trial (NCT02434718), the prodromal or mild AD patients with aducanumab treatment manifested significant reductions of Aβ aggregates and promotion of cognition; however, there were some ARIAs that occurred. Then, 2 phase III studies ENGAGE (NCT02477800) and EMERGE (NCT02484547) were carried on. EMERGE achieved its primary endpoints, but ENGAGE did not. Although some members of US sanitation institutes published opposition to aducanumab for AD treatment, the antibody was still approved by FDA in 2021.
Bapineuzumab	Aβ_42_	Mild to moderate AD	Antibody	Two phase III studies (NCT00667810, NCT00676143) respectively in APOE ε4 carriers and noncarriers all failed to meet the coprimary endpoints.
Donanemab	Aβ_42_	Prodromal and mild AD	Antibody	The phase II trial of donanemab (NCT03367403) showed that a significant reduction of amyloid plaque was detected at 76 weeks of donanemab treatment, and more than half of participants manifested amyloid-negative status at 52 weeks of the treatment. Cognitive and functional decline was slowed down in the donanemab group. Two phase III studies (NCT04437511, NCT05026866) have been conducted to further test the safety and efficacy. Although 24% of participants emerged ARIAs in phase III trials, donanemab was approved by FDA in July 2024.
Lecanemab	Aβ_42_	Prodromal and mild AD	Antibody	The phase II trial of lecanemab (NCT01767311) reported that the treatment resulted in amyloid plaques reduction and sustained clinical remission in AD patients, although the primary endpoint of 12 months was not met. CDR-SB scores of treatment group were significantly lower than control group at 12 months; however, the scores were no difference between the groups at 18 months, which is a confusing result. Two phase III studies (NCT03887455, NCT04468659) have been underway to further test the safety and efficacy. Despite some enigmas unveiled, lecanemab was still approved by FDA in July 2023.
Solanezumab	Aβ_42_	Mild and moderate AD	Antibody	There was no significant cognition improvement observed in mild to moderate AD patients according to the results of the clinical trials of solanezumab (NCT00905372, NCT00904683, NCT01127633, NCT02760602). In the phase II/III study NCT01760005, solanezumab even aggravated the impairment of cognition of dominantly inherited AD patients. The phase III study (NCT02008357) testing the effects of solanezumab on asymptomatic or very mild patients also failed to slow cognitive decline.
Crenezumab	Aβ_42_	Prodromal and mild AD	Antibody	The clinical trials (NCT02670083, NCT03114657, NCT03491150, NCT01998841) failed to hit the endpoints of cognition improvement.
Gantenerumab	Aβ_42_	Prodromal and mild AD	Antibody	The antibody leads Aβ clearance by Fc receptor-mediated phagocytosis. Two phase II clinical trials (NCT04623242, NCT01760005) did not meet the endpoints of cognition improvement, although some indicators of AD declined after administration of the antibody. Recently, 2 randomized, double-blind phase III trials (NCT03444870, NCT03443973) also did not show significant decline of CDR-SB scores after gantenerumab treatment compared with control. Further studies (NCT04339413 and NCT04374253) have been underway to evaluate safety, tolerance, and effectiveness.
Semorinemab	Tau	Mild and moderate AD	Antibody	The phase II trial (NCT02754830) reported that the antibody cannot improve AD symptoms. Another randomized, placebo-controlled, and double-blind phase II Lauriet study (NCT03828747) failed to hit the common primary endpoint of ADCS-ADL.
BIIB076	Tau	AD	Antibody	The phase I trial of the antibody (NCT03056729) has been completed. However, the program was terminated due to “priority ranking of investment portfolio” instead of serious adverse events.
Gosuranemab	Tau	Early AD	Antibody	Gosuranemab (BIIB092) was demonstrated to clean soluble N-terminal tau in CSF (a 98% decrease in the gosuranemab group) in a phase II trial (NCT03068468). Nevertheless, the further phase II trial of the antibody (NCT03352557) was announced failed owing to low effectiveness of cognition mitigation.
Tilavonemab	Tau	Early AD	Antibody	The phase II trial (NCT02880956) failed to demonstrate the effectiveness of cognition improvement.
Bepranemab	Tau	Early AD	Antibody	A phase II study (NCT04867616) is ongoing.
Zagotenemab	Tau	Prodromal and mild AD	Antibody	It failed to hit the primary endpoint according to the result of clinical trial NCT03518073, although its safety was confirmed by 2 phase I trials (NCT02754830, NCT02754830).
Posdinemab	Tau	Prodromal and mild AD	Antibody	The phase I trial NCT03375697. The administration of posdinemab can reduce the level of pTau in the CSF. A phase II study (NCT04619420) is ongoing.
E2814	Tau	Mild AD	Antibody	A phase I/II trial (NCT04971733) is just completed.
Lu AF87908	Tau	AD	Antibody	A phase I trial (NCT04149860) is completed, but the result has not been posted.
RG7345	Tau	AD	Antibody	A phase I trial (NCT04096287) testing safety of PNT001 has been completed. The results showed that single doses of PNT001 were demonstrated as safe and well-tolerated.
PNT001	Tau	AD	Antibody	A phase I trial (NCT04096287) testing safety of PNT001 has been completed. The results showed that single doses of PNT001 were demonstrated as safe and well-tolerated.
AL002	TREM2	Early AD	Antibody	The phase I trial of AL002 (NCT03635047) showed safety and well tolerance. A phase II trial (NCT04592874) is completed, but the result has not been posted.
Daratumumab	CD38	Mild and moderate AD	Antibody	The antibody has been approved by FDA for MM treatment. It is also used to resist the toxicity of CD38^+^CD8^+^ T cells in AD patients. However, no improvement of cognition has been found with daratumumab treatment according to the result of phase II trial (NCT04070378).
Cinpanemab	α-Syn	PD	Antibody	The phase II trial NCT03318523 showed that cinpanemab cannot lead significant change of DaT-SPECT and disease progression.
Prasinezumab	α-Syn	PD	Antibody	Two phase II trials (NCT03100149, NCT04777331) have been active.

AD, Alzheimer’s disease; ADCS-ADL, Alzheimer disease cooperative study–activities of daily living; APOE, apolipoprotein E; ARIAs, amyloid-related imaging abnormalities; Aβ, amyloid β; BACE, β-amyloid precursor protein-cleaving enzyme; CDR-SB, clinical dementia rating–sum of boxes; CSF, cerebrospinal fluid; DaT-SPECT, dopamine transporter single-photon-emission computed tomography; DS, Down syndrome; FDA, Food and Drug Administration; FTD, fast-track designation; hTERT, human telomerase reverse transcriptase; Ig, immunoglobulin; MM, multiple myeloma; MMSE, mini-mental state examination; PD, Parkinson’s disease; TREM2, triggering receptor expressed on myeloid cells 2; α-syn, α-synuclein

#### Alzheimer’s disease

AD is a neurodegenerative disease causing severe, irreversible cognitive decline and progressive disorientation. Although the complete pathogenesis of AD is unclear, amyloid plaques and neurofibrillary tangles (NFTs) are recognized as key pathological hallmarks. They are related to the deposition of β-amyloid peptides, the aggregation of hyperphosphorylated tau proteins, and neuroinflammation [45]. Traditional treatments for AD primarily involve cholinesterase inhibition and antagonism of *N*-methyl-d-aspartate receptors (NMDARs) to restore normal synaptic neurotransmission and memory function [46]. As more critical mechanisms of AD are uncovered, therapies that target AD-related factors through the immune system have gradually become a research hotspot for intervention.

**Fig. 3.**
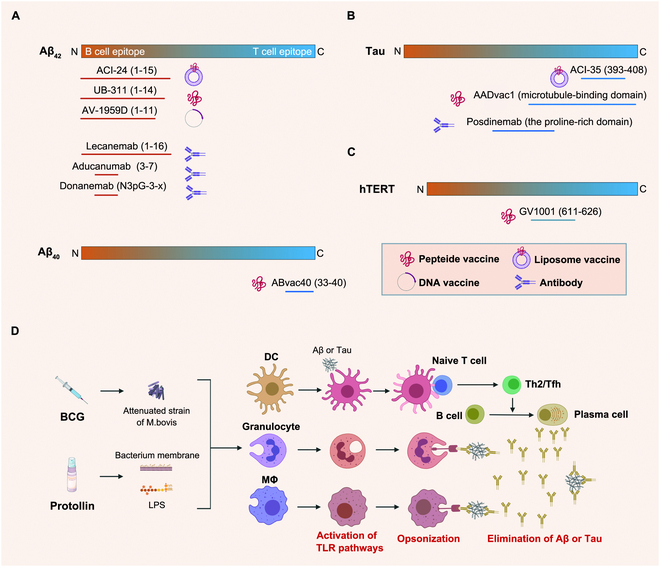
The clinical applied immunotherapies approved by FDA or with positive effects on AD. (A to C) Promising immunotherapies for AD against Aβ (A), Tau (B), and hTERT (C). (D) Approaches to boost innate immunity like BCG and Protollin have been also applied to treat AD. The figure is created with MedPeer (medpeer.cn).

The first attempt of immunotherapy for AD was conducted in 1999, when PDAPP mice immunized with the 42-amino-acid form of Aβ (Aβ_42_) exhibited reduced amyloid plaques, astrocytosis, and neuronal dystrophy. This sparked scientific interest in AD active immunotherapy [47]. To avoid excessive immune activation, it is essential to carefully select immunogens, adjuvants, and carriers to induce moderate immune responses. Immunogen design has focused on the N terminus of Aβ_42_, a B cell epitope with weak T cell immunogenicity activation. Adjuvants like alum, which specifically activate humoral immunity, have replaced QS-21 [48,49]. Ongoing or terminated clinical trials of Aβ_42_ N-terminal vaccines include various vaccines, and most studies have confirmed humoral immune activation through high Aβ_42_ antibody levels in animals and patients [50,51]. Besides Aβ_42_, the 40-amino-acid form of Aβ (Aβ_40_) is also a key target for AD treatment, with related vaccines showing good safety and tolerability [52]. Moreover, several tau aggregate-targeting vaccines have shown efficacy in animal models, with some entering clinical trials [53–55]. Beyond Aβ and tau, vaccines like GV1001, Bacille Calmette–Guérin vaccine (BCG), and Protollin are being explored for potential therapeutic effects by activating innate immunity or other unknown pathways [50]. For detailed information on all AD vaccines, see Fig. 3.

Due to the potential risk of meningoencephalitis, research has gradually shifted to safer passive immunotherapy with antibodies. Monoclonal antibodies (mAbs) currently under clinical evaluation include those targeting Aβ aggregates and tau aggregates [54] (Fig. 3A and B). Positron emission tomography shows that most anti-Aβ antibodies reduce brain Aβ burden, whereas evidence for cognitive benefit from anti-tau antibodies is still limited [56]. In addition to Aβ and tau, certain immune cells are also involved in AD progression and may become therapeutic targets. In early AD patients, CD38^+^CD8^+^ T cells significantly increase in the blood and central nervous system, potentially exerting neurotoxicity [57]. Daratumumab (a CD38 antibody) can eliminate such toxic T cells. In contrast, microglia, as innate immune cells in the central nervous system, exert neuroprotective effects by phagocytosing Aβ. The agonistic antibody AL002 activates microglia by binding to triggering receptor expressed on myeloid cells 2 (TREM2), enhancing their chemotaxis, phagocytosis, lipid metabolism, energy metabolism, and proliferation [58,59]. Phase II data for AL002 and anti-CD38 approaches are pending publication.

Despite significant progress in AD immunotherapy, many mysteries remain regarding the mechanisms of AD and its immunotherapy. First, how Aβ and tau aggregates affect cognition is not fully understood, as some AD patients have no Aβ deposition or have Aβ plaques without cognitive decline [60]. Second, it is necessary to identify the optimal individualized treatment targets, as patients differ in genetic background, environmental exposure, AD staging, and comorbidities, highlighting the need for personalized detection and treatment. Single-cell RNA sequencing (scRNA-seq) and spatial transcriptomics will help accurately identify AD-related factors, and the accumulation of autopsy data can enhance artificial intelligence (AI) prediction and simulation of AD components.

Moreover, neuroinflammation caused by immune system and metabolic changes is crucial in AD progression, and it is necessary to characterize the immune microenvironment in AD patients [61]. Finally, the mechanisms for alleviating the side effects of immunotherapy need to be studied in depth. Although most therapies aim to avoid activating T cell immunity, antibodies from humoral immunity may mediate neurotoxicity through ADCC. Continuous elucidation of the mechanisms of side effects will help reduce the toxicity of immunotherapy.

#### Parkinson’s disease

The pathogenesis of PD is complex, with aggregated α-synuclein playing a crucial role. This protein aggregation triggers a series of events leading to neuronal death and clinical manifestations of the disease. Recent studies indicate that both central and peripheral immune responses are involved in disease progression. Microglia, the resident immune cells of the central nervous system, can be activated into pro-inflammatory phenotypes, exacerbating neuroinflammation and neuronal damage. Peripheral immune cells, such as T cells, may also play a role in the disease process. Some studies have detected autoantibodies and T cells that target α-synuclein in PD patients [62].

Given the central role of α-synuclein, many immunotherapy studies have focused on targeting this protein. mAbs that bind to α-synuclein, such as cinpanemab and prasinezumab, are under clinical trial evaluation, aiming to prevent its aggregation and promote clearance from the brain [63,64]. In a phase 2 trial, cinpanemab showed reductions in CSF α-synuclein aggregates, but no significant clinical benefit was observed in early-stage Parkinson’s patients [63]. Additionally, active immunotherapy strategies are being explored. For example, UB-312 has shown promise in early-phase clinical trials by stimulating the patient’s immune system to produce antibodies against α-synuclein, potentially offering a long-term management solution for PD [65]. The development of conformation-specific vaccines is also a promising direction. These vaccines aim to mimic epitopes present only on the surface of pathological α-synuclein fibrils. Results showed that antibodies produced in immunized mice could recognize α-synuclein fibrils and brain homogenates from Parkinson’s patients [66].

In conclusion, immunotherapy holds promise as a potential disease-modifying therapy for PD, with current research focusing on developing targeted therapies for the underlying pathology of the disease. Methods such as mAb, active immunotherapies, and conformation-specific vaccines are being explored.

### Vascular pathologies

**Fig. 4.**
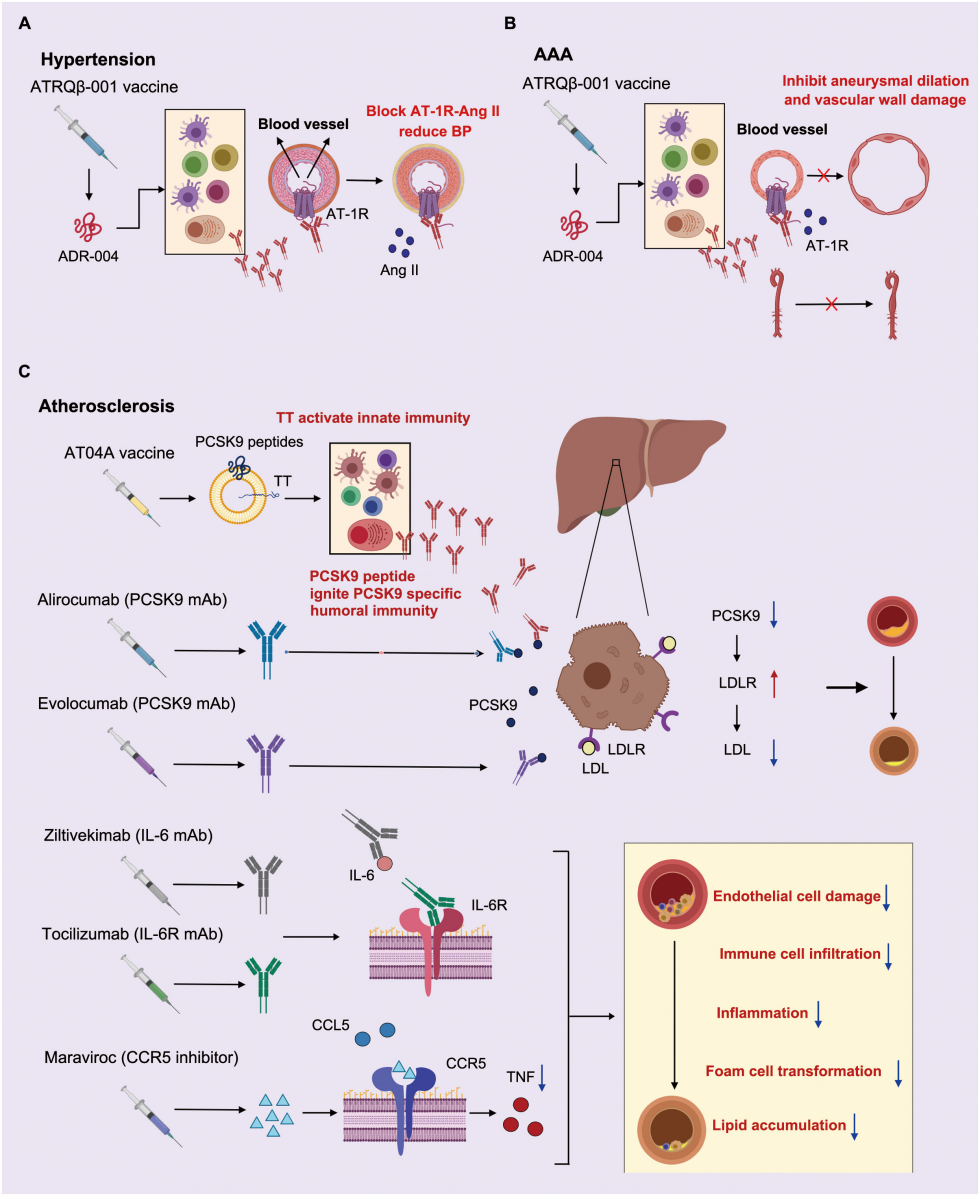
The clinical applied immunotherapies approved by FDA or with positive effects on vascular pathologies. Vaccines, mAb, and small molecules for hypertension (A), AAA (B), and atherosclerosis (C) have been verified as effective to realize blood pressure control, lipid metabolism, and anti-inflammation in clinical trials. The strategies are promising to reestablish a healthy cardiovascular environment in elderly. The figure is created with MedPeer (medpeer.cn).

As age increases, factors like oxidative stress, chronic inflammation, mitochondrial dysfunction, and key pathway dysregulation contribute to the prevalence of vascular pathologies common in the elderly [67]. These accumulating pathologies can lead to strokes, coronary heart disease, and vascular dementia, increasing elderly mortality. Immunotherapies are under investigation to mitigate vascular pathologies. Here, we summarize immunological interventions for hypertension, abdominal aortic aneurysms (AAAs), and atherosclerosis as representative examples (Fig. 4). Related clinical trials are listed in Table 2.

**Table 2.** Summary of clinical trials of the immunotherapies for vascular pathologies

Name	Target	Indication	Modality	Results of clinical trials
PMD-2850	Ang I	Hypertension	Vaccine	In a phase Ia trial, PMD-2850 did not induce the production of antibody against Ang I in healthy people after the first dose.
PMD-3117	Ang I	Hypertension	Vaccine	Single dose of PMD-3117 only mobilized the immune response against KLH in healthy people. Significant immune response to Ang I and blood pressure reduction occurred after second inoculation. However, SADR in phase IIa trial halted further study.
CYT006-AngQb	Ang II	Hypertension	Vaccine	The phase II trial NCT00500786 demonstrated its ability to reduce blood pressure in patients with mild-to-moderate hypertension. Nevertheless, phase III trial was not conducted owing to its relatively modest effectiveness compared to RAS blockers.
AGMG0201	Ang II	Hypertension	Vaccine	The administration of AGMG0201 lowered blood pressure of spontaneously hypertensive rats for 6 months. Well tolerance of the vaccine was shown in patients with essential hypertension in a phase I/IIa study.
ATRQβ-001	A1TR	Hypertension, AAA, AMI, atherosclerosis	Vaccine	Related clinical trials are ongoing.
CETi-1	CETP	Atherosclerosis	Vaccine	Phase II trials with CETi-1 were done to check the safety, tolerance, and immunogenicity in patients with hypercholesterolemia and atherosclerosis in 2004. However, further study did not progress.
ATH03	CETP	Atherosclerosis	Vaccine	The safety, immunogenicity, and dose response were tested in a phase I trial (NCT01284582) in 2012. However, further study did not progress.
V-6	adipose tissue antigens	Atherosclerosis	Vaccine	The phase III trial (NCT03042741) started in 2017, but the result about blood pressure and LDL level have not been available.
AT04A	PCSK9	Atherosclerosis, hypercholesterolemia	Vaccine	Fused PCSK9-TT peptide immunization significantly reduced the level of PCSK9 in plasma of mice. AT04A and AT06A from AFFITOPE® peptide were selected to induce production of anti-PCSK9 antibody in patients with hypercholesterolemia in the phase I study NCT02508896. Only AT04A showed significant LDLc-lowering activity.
PPV	ox-LDL	ACS and atherosclerosis	Vaccine	PPV administration led to a 35% reduction in CVD bed-days and increased the production of anti-ox-LDL. However, there is no significant change in atherosclerosis markers.
Alirocumab	PCSK9	FH, atherosclerosis	Antibody	Alirocumab significantly lowers LDL-C levels by inhibiting PCSK9. In the ODYSSEY OUTCOMES trial, patients achieved an average LDL-C reduction of 48.5%, with some reaching LDL-C levels <25 mg/Dl. In the PACMAN-AMI trial, after 52 weeks of alirocumab combined with statin therapy, PAV in non-infarct-related arteries significantly decreased and MLA increased. The ARCHITECT trial demonstrated that 78 weeks of alirocumab treatment promoted coronary plaque regression and stabilization, particularly in patients with FH. It was approved by FDA in 2015.
Evolocumab	PCSK9	FH, atherosclerosis	Antibody	Evolocumab can significantly decrease the level of LDLc, ApoB, and Lp(a)‌‌. The antibody led significant regression in PAV and reduced microcalcification activity after 18 months of therapy. It was approved by FDA in 2015.
MLDL1278A	ox-LDL, MDA-LDL	Atherosclerosis obesity, T2D	Antibody	It failed to reduce inflammation of artery walls measured by FDG-PET/CT in a phase II trial (NCT01258907).
MEDI6570	LOX-1	AMI, atherosclerosis	Antibody	MEDI6570 was demonstrated as safe and well-tolerated and in phase I trial NCT03654313. Yet, the program was cancelled due to the lack of data confirming the efficiency against atherosclerosis.
Canakinumab	IL-1β	Autoimmune disease, lung cancer, CVD	Antibody	Canakinumab is an FDA-approved agent for CAPS and can also inhibit the lung cancer development via deactivation of inflammasome. In the CANTOS trial (NCT01327846), canakinumab significantly lowered risk of cardiovascular event recurrence in 10,061 patients with previous MI. Yet, it did not change the total mortality owing to the elevation of infection risk.
Xilonix	IL-1α	Advanced gastric cancer, CVD	Antibody	Xilonix has been approved to treat with advanced gastric cancer. In the phase II trial (NCT01270945), the antibody can decrease the incidence of vessel restenosis without significant MACEs at 3-month follow-up of patients after percutaneous revascularization. However, there is no difference between Xilonix and placebo at 12-month follow-up.
Anakinra	IL-1R	RA, NOMID, DIRA, CVD	Antibody	The antibody is an FDA-approved agent for RA, NOMID, and DIRA. Due to the blockade of IL-1 pathway, Anakinra was applied to treat CVD. Clinical trial showed that it reduced hsCRP levels after 14-d treatment with higher risk of MACEs at 1 year in the patients with NSTE-ACS. Another trial reported that Anakinra lowered hsCRP levels and incidence of new-onset heart failure in patients with STEMI.
Tocilizumab	IL-6R	Autoimmune disease, atherosclerosis	Antibody	Tocilizumab can block the IL-6–IL-6R pathway by binding to IL-6R and has been approved to treat different types of arthritis. A phase II trial (NCT03004703) showed its ability to reduce hsCRP in the patients with NSTEMI. It also improved myocardial salvage index and facilitated hsCRP reduction in the patients with STEMI.
Ziltivekimab	IL-6	Atherosclerosis	Antibody	Developed by Novo Nordisk, ziltivekimab was tested in a phase II trial (NCT03926117) in the individuals with chronic kidney disease. The antibody can significantly decrease the level of hsCRP at all doses. The phase III trial of ziltivekimab (NCT05021835) is ongoing.
Adalimumab	TNF-α	Autoimmune disease, atherosclerosis	Antibody	TNF-α was found in atherosclerotic plaques and correlates with CVD. Adalimumab treatment led to reduced blood lipid level, atherogenesis, and lower risk of cardiovascular events in patients with arthritis. It is not suitable for heart failure patients.
Rituximab	CD20	NHL, CLL, RA, atherosclerosis	Antibody	Rituximab is a kind of immunotherapy to eliminate B cells. Research showed that B2 cells accelerate the development of atherosclerosis. The safety has been verified by a clinical trial (NCT03072199) in the patients with acute STEMI.
Plozalizumab	CCR2	Melanoma, atherosclerosis	Antibody	The phase II trial demonstrated that plozalizumab reduced the level of hsCRP and the risk of atherosclerosis. Due to lack of data supporting the efficacy for atherosclerosis, the program was withdrawn.
Maraviroc	CCR5	HIV, atherosclerosis	Small molecule	Maraviroc is an FDA-approved agent for HIV. In mouse model and preliminary data in humans, maraviroc showed anti-atherosclerotic effect. Further phase IV trial (NCT03402815) is ongoing.
AZD5069	CXCR2	CVD, COPD	Small molecule	AZD5069 administration led the reduction of neutrophils. The phase II trial (ISRCTN48328178) of the agent was conducted to evaluate improvement of CVD in the patients undergoing cutaneous coronary intervention.
POL6326	CXCR4	CVD	Small molecule	A phase II clinical trial (NCT01905475) has been completed, but the result is still unavailable.
Colchicine	Immunocytes	Autoimmune disease, CVD	Small molecule	Colchicine deactivates inflammation via blocking cytoskeletal microtubule formation. In the LoDoCo2 trial, patients with stable chronic CAD treated with colchicine had a 31% reduction in the incidence of cardiovascular events. Another phase III trial (COLCOT) verified its function in patients with MI.
Methotrexate	Immunocytes	Autoimmune disease, CVD	Small molecule	Methotrexate inhibits the proliferation of immunocytes by binding to DHFR. A clinical trial (NCT04616872) was conducted to evaluate the improvement of CVD of methotrexate loading by LDL-like NPs in patients with multivessel CAD. The result showed that plaque volume was decreased by the agent.
Dexamethasone	Immunocytes	Autoimmune disease, CVD	Small molecule	A phase II clinical trial (NCT01983449) has shown that dexamethasone injection reduced restenosis at a year follow-up in the patients with symptomatic PAD receiving PTA or atherectomy.
Hydroxychloroquine	Immunocytes	Malarial, SLE, CVD	Small molecule	A cohort study revealed that hydroxychloroquine treatment can decrease CAD risk in RA patients. Then, 2 phase IV trials (NCT02648464, NCT02874287) began to test the safety and the ability to control hsCRP level.
Paclitaxel	T cell	Cancers, CVD	Small molecule	The clinical trial (NCT04148833) testing paclitaxel’s effect on plaque volume in patients with CAD is ongoing.
Proleukin	Treg	Autoimmune disease, atherosclerosis	Cytokine	The affinity of CD25 (IL-2Rα) to IL-2 is much higher than IL-2Rβ and IL-2Rγ, leading Treg (highly express CD25) stimulated by IL-2 in a low dose. Low-dose IL-2 was tested in CVD, and its safety, tolerability, and anti-inflammation effect have been verified (NCT03113773, NCT04241601).

ACS, acute coronary syndrome; AMI, acute myocardial infarction; Ang I, angiotensin I; Ang II, angiotensin II; ApoB, apolipoprotein B; AT1R, angiotensin II type 1 receptor; CAD, coronary artery disease; CAPS, cryopyrin-associated periodic syndromes; CCL, chemokine (C-C motif) ligand; CCR, chemokine (C-C motif) receptor; CETP, cholesteryl ester transfer protein; CLL, chronic lymphocyte leukemia; COPD, chronic obstructive pulmonary disease; CVD, cardiovascular disease; CXCR, C-X-C motif chemokine receptor; DHFR, dihydrofolate reductase; DIRA, deficiency of the interleukin-1 receptor antagonist; FDA, Food and Drug Administration; FDG, fluorine-18 fluorodeoxyglucose; FH, familial hypercholesterolemia; HIV, human immunodeficiency virus; IL, interleukin; hsCRP, hypersensitive C-reactive protein; KLH, keyhole limpet hemocyanin; LDL, low-density lipoprotein; LDLc, low-density lipoprotein cholesterol; LOX1, lectin like oxidized low-density lipoprotein receptor 1; Lp(a), lipoprotein(a); mAb, monoclonal antibody; MACEs, major adverse cardiovascular events; MAPK, mitogen-activated protein kinase; MDA, malondialdehyde; MI, myocardial infarction; NHL, non-Hodgkin lymphoma; MLA, minimum lumen area; NOMID, neonatal onset multisystem inflammatory disease; NPs, nanoparticles; NSTE, non-ST elevation; NSTEMI, non-ST-elevated myocardial infarction; ox-LDL, oxidized low-density lipoprotein; PAD, peripheral arterial disease; PAV, plaque volume; PET/CT, positron emission tomography-computed tomography; PPV, pneumococcal polysaccharide vaccine; PTA, percutaneous transluminal angioplasty; RA, rheumatoid arthritis; RAS, renin-angiotensin system; SADR, serious adverse drug reaction; SLE, systemic lupus erythematosus; STEMI, ST-elevated myocardial infarction; T2D, type 2 diabetes; TNF, tumor necrosis factor; Treg, regulatory T cell; TT, tetanus toxoid

#### Hypertension

Hypertension, characterized by sustained blood pressure above 130/80 mmHg, is a major health threat, particularly in the elderly. Traditionally, overactivation of the sympathetic nervous system and the renin–angiotensin–aldosterone system (RAAS) is considered a key contributor of hypertension. In addition to chemical antagonists, immunotherapies are being used to block related receptors in the systems.

In the first 2 decades of the 21st century, the vaccines targeting the RAAS were tested in animals and clinical trials. For example, the vaccine ATRQβ-001, targeting the angiotensin II type 1 receptor (AT1R), showed high efficiency in resisting hypertension and preventing various cardiovascular diseases in animal models [68]. However, these vaccines’ long half-life and ability to induce RAAS-targeting antibodies may affect blood pressure stability [69]. Additionally, vaccines targeting the sympathetic nervous system have shown potential in hypertension treatment, such as selecting endothelin-A receptors (ETARs) and α1-adrenergic receptors (α1-ARs) as immunogens to regulate vessels and nerves, thereby combating pulmonary and aortic hypertension in animal models [70,71]. Beyond active immunization, key factors in the sympathetic nervous system and RAAS can also be blocked via passive immunization. For instance, mAb against the C terminus of human angiotensin-(1–12) can lower arterial pressure in humanized rat models [72]. Another research group focused on using antibodies to block G protein-coupled receptors (GPCRs) to alleviate hypertension, screening and modifying humanized nanobodies like AT118i4 as AT1R antagonists, offering new therapeutic avenues [73].

Despite the great success of angiotensin-converting enzyme inhibitors (ACEIs) and angiotensin II receptor blockers in hypertension treatment, about 40% of patients still fail to normalize their blood pressure according to the results of related clinical trials [74]. Over the past decade, increasing evidence indicates that alterations in the immune system may contribute to the development of hypertension [75]. In detail, various environmental and lifestyle factors facilitate the formation of damage-associated molecular patterns (DAMPs) and hypertension-related neo-antigens, leading to antigen-presenting cell (APC) activation and neoantigen presentation. The mobilization of innate immunity triggers adaptive immune responses against vasculature, including CTL cytotoxicity, increased central memory CD8^+^ T cells, plasma cell-derived autoantibodies, and elevated pro-inflammatory cytokines. On the contrary, immunosuppressive cells like invariant NK T (iNKT) cells, regulatory T cells (Tregs), myeloid-derived suppressor cells (MDSCs), and CD4^+^ choline acetyltransferase (ChAT)^+^ T cells protect against vascular damage by expressing anti-inflammatory factors. In particular, CD4^+^ChAT^+^ T cells not only inhibit the generation and function of Th1, Th17, γδT cells, and CD8^+^T cells but also exert vasodilatory effects via cholinergic neuroregulation [76]. The cycles of damage and repair result in vascular collagen deposition and fibrosis, accelerating vascular sclerosis and dysfunction [75]. More studies need to be conducted to clarify the precise markers of related immune cells and find optimal dose for minimal adverse events.

Additionally, hypertension is more prevalent among the elderly and is associated with aging-related dysfunction of the sympathetic nervous system, the RAAS, and chronic inflammation. Immunotherapies to clear senescent cells and block SASPs may slow the progression of hypertension, but further research is needed to verify their efficacy and safety.

#### Abdominal aortic aneurysms

AAA, characterized by an abdominal aortic diameter enlargement to 1.5 times normal or reaching 3 cm, can cause sudden death due to aortic rupture and hemorrhage. The incidence of AAA increases with age, making ruptured AAA an important, though uncommon, cause of sudden death in older adults. However, effective therapies for AAA remain scarce, and long-term survival rates remain low even after surgical intervention [77]. In recent decades, the abnormality of the immune system and chronic inflammation is critical in AAA progression. Infiltration of M1 macrophages, neutrophils, and lymphocytes into vessels mediates vascular smooth muscle cell (VSMC) apoptosis and extracellular matrix (ECM) degradation via immune signaling activation and pro-inflammatory cytokine secretion [77]. This establishes an inflammatory microenvironment and promotes AAA development. Additionally, angiotensin II, a regulator of blood pressure, has been shown to exacerbate vascular inflammation by influencing macrophage polarization, increasing matrix metalloproteinase 9 (MMP9) expression, and promoting the differentiation of hematopoietic stem and progenitor cells (HSPCs) into myeloid cells [78–80]. These insights into AAA pathogenesis have laid the foundation for developing new therapeutic strategies.

Although immunotherapies are not yet widely accepted as clinical treatments for AAAs, numerous studies have explored them as alternatives to suppress AAA formation and progression. Theoretically, rebalancing pro-inflammatory factors (e.g., MMP-2, MMP-9, IL-1β, IL-6, IL-17, TNF-α, CCL2, and CCL3) and anti-inflammatory factors (e.g., IL-4 and IL-10) can reshape the inflammatory microenvironment and improve the condition of AAA. However, given the multi-system roles of cytokines and chemokines, blocking or adding them may cause off-target immune effects [81,82]. Beyond targeting the AAA microenvironment, vaccines against angiotensin II (Ang II)–AT1R pathway have shown preventive effects against AAA initiation in rodent models by blocking vascular growth and inflammation [50]. In a rat model, Kurashiki et al. [83] immunized rats with Ang II peptides conjugated to KLH, and the vaccine significantly reduced macrophage infiltration and inflammatory responses in AAA walls by inhibiting nuclear factor κB (NF-κB) and c-Jun N-terminal kinase (JNK) signaling. Another vaccine, ATRQβ-001, targeting AT1R, has also been shown to prevent AAA initiation and progression in mice [84]. To date, no immunotherapy has demonstrated significant clinical efficacy for AAA.

#### Atherosclerosis

As the major cause of cardiovascular disease, atherosclerosis is a chronic vascular lesion resulting from the increase of cholesterol-containing low-density lipoprotein (LDL) particle in the arterial wall, which can be induced and augmented by genetic factors, exposure, and metabolic disorder [85]. Inflammatory immune cells and cytokines have been identified as key contributors to the formation and enlargement of atherosclerotic plaques [86]. Briefly, monocyte-derived macrophages, VSMCs, and resident immunocyte ingest lipoprotein to form foam cells, creating plaque necrotic cores [87]. Lipid antigens in plaque, including apolipoprotein B (ApoB) and cholesterol crystals, enhance innate immunity, promoting inflammatory and chemokine production and driving atherosclerosis progression [87,88]. Beyond inflammation, aging contributes significantly to the progression of atherosclerosis. Senescent VECs cause vasoconstriction and inflammation via increased endothelin and ROS [89]. Senescent VSMCs reduce mechanosensation and ECM remodeling, leading to arterial stiffness and vascular microenvironment changes [90]. Simultaneously, immunosenescence causes immune metabolic and functional disorders, increasing myeloid-derived immune cells and activating innate immunity [91]. These factors together create a unique microenvironment conducive to atherosclerosis.

According to the pathogenesis, many immunotherapies have been designed to target different key factors involved in atherosclerosis. Immunization against LDL has shown anti-atherosclerotic effects in animal models, pioneering atherosclerosis immunotherapy research [92]. However, cardiovascular benefits in clinical trials have been limited. Strategies targeting oxidized LDL (ox-LDL), ApoB, phospholipase A2 (PLA2), lectin like oxidized low-density lipoprotein receptor 1 (LOX1), proprotein convertase subtilisin/kexin type 9 (PCSK9), cholera toxin B subunit (CTB), and a disintegrin and metalloproteinase with thrombospondin type 1 motif 7 (ADAMTS7) through vaccination, antibodies, or inhibitors have further confirmed the effectiveness of eliminating lipoprotein abnormalities [50]. Among these agents, evolocumab and alirocumab, targeting PCSK9, exhibit high efficiency to lower cholesterol and inhibit the progression of atherosclerosis [93,94].

Direct interventions to the vascular immune microenvironment can also reduce the incidence of atherosclerosis and slow the prognosis, such as using broad-spectrum immunosuppressants and precise immunomodulation targeting specific molecules. For example, canakinumab, a mAb against IL-1β, can lower recurrent cardiovascular event rates but failed to decrease the total mortality because of elevated risk of infection after the antibody administration [95]. Subsequent studies have developed more specific anti-IL-1α mAbs and IL-1 receptor antagonists to reduce atherosclerotic inflammation. However, the impact of IL-1 receptor antagonists on atherosclerosis, mortality, and cardiovascular adverse events requires further study [96]. Moreover, IL-6 antagonists have been used to suppress atherosclerotic inflammation and have shown efficacy in cardiovascular disease related to ST-elevated myocardial infarction and chronic kidney disease [97]. A phase II trial of IL-6 antibody ziltivekimab showed that IL-6 blockade reduced median high-sensitivity C-reactive protein (hs-CRP) by more than three-quarters in patients at high atherosclerotic risk, indicating the potential of the therapy [98].

In addition to cytokines, chemokines are also therapeutic targets for atherosclerosis. Blocking related chemokines can inhibit inflammatory immunocyte recruitment, especially macrophages [88]. Additionally, balancing the adaptive immune response is effective to improve vascular lesion. This can be achieved by inhibiting macrophage, effector T and B cell functions via proliferation inhibitors, costimulatory molecule antibodies, and B cell depletion, or by increasing Treg numbers and function with low-dose IL-2 and CD27 agonists [87,99].

### Type 2 diabetes

T2D is a chronic metabolic and inflammatory disease linked to aging. It is characterized by a triad of β cell dysfunction, insulin resistance, and chronic low-grade inflammation, resulting in persistent hyperglycemia and related complications of cardiovascular and central nervous system [100]. Traditional treatments for T2D focus on the control of carbohydrate metabolism and the protection of β cells. However, as the pathogenesis of T2D becomes clearer, immunotherapy has emerged as a potential alternative treatment. Immunotherapeutic approaches mainly focus on 3 aspects: metabolic regulation, β cell protection, and anti-inflammatory treatment.

The promotion of carbohydrate metabolism is one of the key strategies for T2D treatments. Glucagon-like peptide-1 (GLP-1) and glucose-dependent insulinotropic polypeptide (GIP) play an essential role in maintaining glucose homeostasis via elevating the production and sensitivity of insulin, which can be degraded by dipeptidyl-peptidase 4 (DPP4) [101,102]. With aging, increased expression of DPP4 destroys the balance of carbohydrate metabolism [103]. A therapeutic vaccine developed by Pang et al. [104], which couples the DPP4 E3 region with KLH, has significantly reduced the level of blood glucose and up-regulated plasma insulin and pancreatic insulin content in HFD-fed mice and KK-Ay and db/db mouse strains without serious adverse events. Additionally, the metabolic regulator adiponectin, secreted by adipocytes, improves metabolism by activating AMPK and peroxisome proliferator-activated receptor-α (PPAR-α) [105]. It can also induce the browning of subcutaneous adipose tissue and improve carbohydrate metabolism by facilitating M2 macrophage polarization [106]. The antibodies that activate adiponectin receptor (AdipoR) have significantly reduced plasma glucose levels and insulin resistance in HFD mice, indicating the feasibility of immunotherapy against AdipoR [107]. Besides, immunotherapies against other metabolic molecules such as calcitonin gene-related peptide and prorenin have also shown the potential for treating diabetes by improving metabolism [108,109].

Protecting β cells is another therapeutic strategy for T2D. Misfolded aggregates of islet amyloid polypeptide (IAPP) may contribute to β cell failure and the development of T2D [110]. It is shown that the overexpression of IAPP reshapes the islet transcriptome, characterized by the down-regulation of β cell identity genes and the up-regulation of inflammation-related gene expression [111]. In 2020, Roesti et al. [112] designed a virus-like particle (VLP) vaccine against the N terminus of IAPP, which inhibited IAPP fibril formation by inducing high-affinity antibodies in mice, which have low affinity for soluble IAPP and do not interfere with its physiological function. Studies have also shown that mAb generated from biopsies of T2D patients, which recognize pathogenic IAPP aggregates, prevent hyperglycemia and IAPP fibril formation in mouse model [113]. Furthermore, immunizing mice with human IAPP fibril immunogens stabilized by nucleobindin 1 technology has generated specific mAb against human IAPP fibrils, which can extend the survival time of diabetic mice [114].

Chronic inflammation, especially in elderly, plays a crucial role in metabolic disorder and β cell damage. The activation of the NLRP3 inflammasome and the maturation of IL-1β have been shown to mediate key pro-inflammatory effects [115]. Canakinumab, a Food and Drug Administration (FDA)-approved IL-1β mAb, can dramatically reduce the level of inflammatory markers, such as hs-CRP and IL-6, but has not been effective in reducing the incidence of T2D [116]. In contrast, an IL-1β vaccine carrying VLPs has shown improvement in blood glucose and CRP levels in a preclinical and clinical study, although long-term effects remain to be evaluated [117]. It should be noted that long-term blockade of IL-1β by immunotherapy may jeopardize the normal immune function and elevate the risk of severe upper respiratory infections, highlighting the importance of determining the optimal dose and dosing schedule [118].

### Arthritis

Arthritis, generally classified into rheumatoid arthritis (RA) and osteoarthritis (OA), is characterized by joint inflammation and surrounding tissue damage, leading to chronic pain, disability, and functional limitations. It remains a leading cause of global disability with significant healthcare costs. The elderly population exhibits a high prevalence of arthritis due to accumulation of senescent cells, immunosenescence, inflammaging, metabolic and endocrine disorders, and intestinal dysbacteriosis. Immunotherapy has gained great success to treat arthritis by remodeling of inflammatory immune microenvironment over the past 2 decades. In this part, we will generate the current mainstream and novel strategies of immunotherapy against RA and OA.

#### Rheumatoid arthritis

RA is a chronic autoimmune disease characterized by synovial inflammation and joint destruction, with a global prevalence of 0.5% to 3% [119]. Its pathogenesis involves complex interactions among genetic predisposition, environmental factors (e.g., smoking), and immune dysregulation, leading to the production of anti-citrullinated protein antibodies, synovial inflammatory cell infiltration (e.g., macrophages, T cells, and B cells), and excessive release of pro-inflammatory cytokines (e.g., TNF-α, IL-1β, and IL-6), ultimately resulting in joint damage [120]. Traditional clinical management of RA primarily consists of nonsteroidal anti-inflammatory drugs (NSAIDs) and conventional synthetic disease-modifying antirheumatic drugs (cDMARDs; e.g., methotrexate). However, these agents remain associated with systemic adverse effects (e.g., hepatotoxicity and infection risk), frequent administration, long-term drug tolerance, and high costs [121]. Approximately 5% to 20% of patients are unresponsive or intolerant to current therapies, underscoring the urgent need for more precise and efficient treatment strategies.

The development of antibody engineering revolutionizes the immunotherapy for RA. Biologic DMARDs (bDMARDs) have gained great success in treating RA, including TNF-α inhibitors (e.g., infliximab), IL-6 receptor blockers (tocilizumab), and B cell-depleting agents (rituximab). Emerging evidence supports abatacept [CTL-associated antigen-4 (CTLA-4)–Ig] for intercepting pre-RA stages by modulating T cell costimulation, demonstrating reduced progression from autoantibody-positive at-risk states to clinical RA [122]. Next to bDMARDs, vaccines carrying rapamycin and peptides from RA-related antigens have been proven to alleviate RA by inducing immune tolerance. DR4-AL179, the vaccine loaded with the immunodominant type II collagen COL2 peptide (259–273) that binds to major histocompatibility complex II (MHC), exhibited robust effectiveness in suppressing arthritis progression in mouse model of RA [123]. Another vaccine against citrullinated antigen was observed to ameliorate RA by down-regulating inflammatory Th cells and cytokines, and dampening the dysregulated V(D)J recombination in mouse model [124]. Compared to immune tolerance induction, adoptive transfer of immunocytes including regulatory dendritic cells (DCs), Treg, and CAR-T against B cells can directly improve the inflammatory microenvironment in RA [125–127]. Recent studies highlight mesenchymal stem cells (MSCs) as promising therapeutic agents for RA due to their dual immunomodulatory and tissue repair capacities [128]. Nevertheless, their low efficacy and unpredicted side effects restrict the clinical application. Many efforts have been made to elevate the efficacy and safety by genetic engineering, hypoxia-activated nanoagent assistance, photothermal therapy combination, and MSC-derived extracellular vesicle (EV) therapy [129].

In summary, immunotherapy has represented promising potential in RA treatment. Future directions of related research encompass the integration of gene-editing technologies, the development of smart stimuli-responsive materials, and the systematic integration of multi-omics data to decipher the immune microenvironment. These advancements aim to design precision-tailored therapeutic regimens for RA.

#### Osteoarthritis

OA, a prevalent degenerative joint disease intricately related to aging, affects various joints including the knee, hip, ankle, hand, and spine, significantly impacting patients’ quality of life. Its pathogenesis centers on progressive cartilage degradation, accompanied by subchondral bone remodeling, chondrocyte-mediated disruption of the synovial microenvironment, and chronic low-grade inflammation driven by immunosenescence and increased SASPs [130,131]. Inflammatory cytokines, including TNF, IL-1, IL-6, and IL-17, exert significant effects on bone and joint deterioration, while some chemokines such as CCL2, CCL3, and CCL4 facilitate the infiltration of inflammatory immune cells into the joint, exacerbating the OA condition [132,133]. Although analgesics can provide short-term symptom relief, they cannot reverse the pathological process, and surgical risks are particularly high for elderly patients, especially those of advanced age [134].

Immunotherapy, which modulates the immune state of patients, has enhanced the treatment of OA and has emerged as a promising therapeutic approach. NSAIDs, as basic therapy, can suppress joint inflammation but are associated with individual variability and cardiovascular risks [135]. Cytokine blockers (e.g., diacerein and adalimumab) can alleviate pain but have not been effective in slowing disease progression, possibly due to the complex signaling pathways in OA. Given this, the combination of multiple cytokine inhibitions are being tested to enhance efficacy. Adoptive transfer of anti-inflammatory cells has also been proven to more effectively relieve OA. MSC therapy has shown potential by secreting immunosuppressive factors such as indoleamine-2,3-dioxygenase (IDO), prostaglandin E2 (PGE2), TGF-β, IL-10, and PD-L1, and working synergistically with exosomes to promote M2 macrophage polarization, inhibit T cells, and induce regulatory B cells [136,137]. Phase I/II clinical trials have confirmed that bone marrow/adipose-derived MSCs can improve cartilage thickness and provide analgesia [138,139]. Specifically, mononuclear cells from blood or spleen are induced into chondrogenic-activated T cells (termed as CATs) in activation media containing chondrocyte lysates and immunosuppressants. A coculture protocol of CATs with adipose-derived MSCs has shown reparative and anti-inflammatory effects on synovial cartilage in preclinical models [140].

### Osteoporosis

Osteoporosis, a systemic skeletal disorder, is characterized by progressive reduction in bone mineral density and deterioration of bone microarchitecture, collectively contributing to elevated bone fragility and fracture susceptibility. The pathogenesis of this condition primarily stems from an imbalance in bone remodeling, where osteoclast-mediated bone resorption chronically exceeds osteoblast-mediated bone formation. This imbalance is further compounded by age-related physiological changes such as hormone disorder, cellular senescence, and chronic low-grade inflammation [141]. Osteoporosis predominantly affects older populations, especially post-menopausal women and men aged 65 years and above, in whom age-related bone loss accelerates [142].

The immunotherapies targeting key factors of osteogenesis and osteoclasis have been demonstrated effective in alleviating osteoporosis. Denosumab, a fully humanized mAb, exerts its therapeutic effect by binding to the receptor activator of NF-κB ligand (RANKL)—the pivotal molecular mediator governing osteoclastogenesis and osteoclastic activation [143]. As the first FDA-approved mAb for osteoporosis, denosumab has shown anti-fracture efficacy comparable to bisphosphonates, with 50% to 70% reduction in vertebral fractures and 40% reduction in hip fractures [144]. However, the mAb exhibits complete pharmacological reversibility upon treatment discontinuation [145]. This characteristic necessitates careful clinical management, as abrupt cessation without subsequent sequential antiresorptive therapy may result in a rapid rebound increase in bone resorption markers. Romosozumab, another FDA-approved mAb, represents a dual-action anabolic therapy for osteoporosis. It selectively binds to sclerostin, stimulating bone formation and simultaneously inhibiting bone resorption through Wnt signaling pathway modulation [144]. Clinical trials have demonstrated its superior anti-fracture efficacy compared to traditional anti-resorptives, with significant increases in bone mineral density (BMD) [146]. Nevertheless, a phase 3 trial revealed potential cardiovascular risks including myocardial infarction [147]. Like denosumab, the transient anabolic effects of romosozumab necessitate sequential therapy with anti-resorptive agents like bisphosphonates to maintain BMD gains [144,145].

### Chronic obstructive pulmonary disease

COPD, the world’s third leading cause of death (WHO, 2023), is a progressive and debilitating respiratory condition marked by chronic airway inflammation, alveolar-capillary unit failure, and declining lung function. Aging is a significant risk factor for COPD as it leads to alterations in the compartments containing respiratory epithelium, lung progenitor cells, pulmonary immune cells, and the interstitium, thus impacting the structure and function of the respiratory system. Molecular mechanism studies showed that enhanced SASPs such as IL-6 and IL-8 induced by inflammaging aggravate the damage of lung tissue [148]. Cellular senescence contributes to dysfunction of lung cells and stem cell exhaustion that decreases the regenerative capacity [149]. Furthermore, mitochondrial dysfunction caused by aging aggravates oxidative stress, promoting DNA damage and DAMP release, thereby driving inflammation. Thus, inhibiting inflammation and anti-aging are central to COPD treatment [150].

Immunotherapies for COPD are not yet widely used clinically. Recent clinical trials showed that triple combinations such as fluticasone furoate/umeclidinium/vilanterol (FF/UMEC/VI) can significantly lower the rate of moderate-to-severe acute exacerbations in COPD patients by inhibiting immune response, yet more research is needed to confirm their efficacy [151]. Beyond corticosteroid, resveratrol, a plant-derived anti-inflammatory agent, has proven effective in relieving COPD symptoms [149]. In animal models, resveratrol down-regulated the production of IL-6, IL-8, IL-17, TNF-α, etc., and mitigated oxidative and fibrotic processes, revealing its anti-aging properties [152,153]. Blocking the type 2 inflammatory pathway is also a promising strategy. For example, dupilumab, an mAb against IL-4Rα, can block IL-4 and IL-13 signaling to reduce eosinophil activation, thereby attenuating type 2 inflammation in patients with COPD. Phase III trials BOREAS and NOTUS demonstrated that dupilumab significantly reduces exacerbation rates and markedly improves lung function in COPD patients, with a favorable safety profile [154]. Approved by the European Medicines Agency (EMA) in July 2024, it stands as the world’s first biologic therapy specifically targeted for COPD. Other mAbs against IL-4, IL-13, IL-33, IL-5, and thymic stromal lymphopoietin (TSLP) have also been tested in clinical trials to explore its efficacy and safety [155–158] (Table 3). Besides, some novel assembling immunosuppressive agents have been demonstrated to reduce inflammation and improve lung function in an animal model of COPD. Guo et al. [159] developed poly(lactic-co-glycolic acid) nanoparticles (PLGA NPs) loading ONX-0914 (an immunoproteasome-specific inhibitor) to alleviate airway inflammation and improve lung function by macrophage polarization inhibition through interferon regulatory factor 4 (IRF4), nuclear factor erythroid 2-related factor-1 (NRF1), and NRF2–p62 axis. Future research will focus on identifying novel type 2 inflammatory biomarkers and non-type 2 inflammatory targets.

**Table 3.** Summary of clinical trials of the mAbs for COPD

Name	Target	Sponsor	Indication	Latest status of clinical trials	ClinicalTrials.gov number
Dupilumab	IL-4Rα	Regeneron/Sanofi	Moderate or severe COPD	Phase III-complete (BOREAS) (2023) Phase III-completed (NOTUS) (2024) Approved by the EMA in July 2024	NCT03930732 NCT04456673
Stapokibar (CM310)	IL-4Rα	Keymed Biosciences Inc./JMT-BIO/CSPC	Moderate or severe COPD	Phase II and III-not yet recruiting	NCT06547333
SSGJ-611	IL-4Rα	Sunshine Guojian Pharmaceutical	Moderate or severe COPD	Phase III-not yet recruiting	Not in ClinicalTrials
APG808	IL-4Rα	Apogee Inc	Mild or moderate COPD	Phase Ib-ongoing	Not in ClinicalTrials
Lebrikizumab	IL-13	Demira Inc./Tanox Inc.	COPD and a history of exacerbations	Phase II-complete (2016)	NCT02546700
Tezepelumab	TSLP	Amgen/AstraZeneca	Moderate to very severe COPD	Phase IIa-complete (2024) Phase IIa-recruiting Phase III-recruiting Phase III-recruiting	NCT04039113 NCT05507242 NCT06878261 NCT06883305
Ecleralimab (CSJ117)	TSLP	Novartis	COPD	Phase IIa-complete (2022)	NCT04882124
BSI045B	TSLP	Biosion Inc./NJCTTQ	COPD	Phase II-recruiting	NCT06707883
QX008N	TSLP	Qyuns Therapeutics Co. Ltd./Joincare Pharmaceutical Group Industry Co. Ltd.	COPD	Phase Ib-ongoing	Not in ClinicalTrials
Tozorakimab	IL-33	AstraZeneca	COPD and chronic bronchitis	Phase I-complete (2024) Phase III-active Phase II-recruiting	NCT06304961 NCT06040086 NCT06897748
Mepolizumab	IL-5	GlaxoSmithKline	COPD	Phase III-complete (2024) Phase III-complete (2024) Phase III-active	NCT04133909 NCT04133909 NCT05138250
Benralizumab	IL-5RA	AstraZeneca	COPD	Phase III-complete (2018) Phase III-active	NCT02155660 NCT04053634

COPD, chronic obstructive pulmonary disease; CSPC, CSPC Pharmaceutical Holdings Group Co. Ltd.; EMA, European Medicines Agency; JMT-BIO, Shanghai Jinmante Biotechnology Co. Ltd.; mAb, monoclonal antibody; NJCTTQ, Nanjing Chia-Tai Tianqing Pharmaceutical Company; TSLP, thymic stromal lymphopoietin

### Fibrosis

Characterized by aberrant accumulation of ECM, fibrosis results from dysregulated post-injury repair responses and can progress to multi-organ failure. Epidemiological and molecular studies indicate that increased senescent cells, SASP levels, and inflammation are primary causes of fibrosis [160]. During aging, rising damaged cells and declining immunosurveillance lead to senescent cell accumulation. SASP from these cells promotes fibroblast and myofibroblast proliferation/transformation, boosting ECM protein synthesis and causing organ fibrosis [161]. The TGF-β1/Smad pathway impacts multiple downstream pathways, including mitogen-activated protein kinase (MAPK), phosphatidylinositol 3-kinase (PI3K)/AKT/mammalian target of rapamycin (mTOR), p53, and Wnt/β-catenin, thereby inducing fibrosis via inflammation (e.g., activating the IL-17A/IL-1β/TGF-β1 axis) [162]. Aging-related ROS increases further activate TGF-β signaling, creating a positive feedback loop. Moreover, integrins and chemokines play key roles in fibrosis development by mediating cell–ECM interactions and immune responses, directly binding to ECM components, and controlling its remodeling [160].

Fibrosis is usually caused by autoimmune and metabolic diseases like systemic sclerosis, autoimmune hepatitis, nonalcoholic steatohepatitis (NASH), nephritis, and Dupuytren’s disease. Clinically, corticosteroids and chemotherapeutic immunosuppressants are used to alleviate symptoms, but their effectiveness is limited. Recently, specific blocking of pro-fibrotic factors has shown potential. Antibodies neutralizing TGF-β, IL-4, IL-13, IL-1, IL-6, TNF-α, CCL2, and chemokine (C-C motif) receptor 2 (CCR2)/CCR5 have been evaluated for efficacy in clinical trials, with some combination blockades showing promise [160]. All the related clinical trials of immunotherapies are listed in Table 4.

**Table 4.** Summary of clinical trials of the immunotherapies for fibrosis

Name	Target	Indication	Modality	Results of clinical trials
Metelimumab (CAT-192)	TGF-β1	Early-stage dcSSc	Antibody	CAT-192 was granted ODD for SSc by FDA. However, related clinical trials had been halted due to serious adverse events and low efficiency of CAT-192 to improve MRSS.
Fresolimumab (GC1008)	All types of TGF-β	SSc	Antibody	In 2005, the antibody was tested in IPF patients in a phase I trial (NCT00125385); however, the result remains unpublished. The phase I trial (NCT01291784) of fresolimumab for myelofibrosis was terminated owing to its cardiotoxicity. Furthermore, the antibody also failed to meet the primary or secondary endpoints in treatment of SR-FSGS. Another small open-label trial (NCT01284322), fresolimumab significantly decreased the expression of THBS1 and COMP, and improved mRSS in SSc patients. However, no follow-up study has been reported.
Dactrekumab (QAX576)	IL-13	IPF	Antibody	The phase II trial (NCT00532233) evaluating the effect of dactrekumab in IPF patients discontinued prematurely due to slow participant enrollment. In 2015, a phase II clinical study was conducted to evaluate the safety, tolerability, pharmacokinetics, and efficacy of dactrekumab in patients with rapidly progressive IPF (NCT01266135, phase II). The study enrolled 40 patients who were randomly assigned to receive intravenous injections of 10 mg/kg dactrekumab at 4-week intervals. The trial was halted after an interim analysis suggested that it was unlikely to meet its endpoints.
Tralokinumab	IL-13	IPF	Antibody	Animal experiments have shown that tralokinumab significantly reduces fibrotic markers. A phase II trial (NCT01629667) was conducted to evaluate safety and efficiency. However, the study was halted due to the high dropout rate and low effect.
Lebrikizumab (TNX-650)	IL-13	IPF	Antibody	A phase II trial (NCT01872689) evaluated the efficacy and safety of the mAb in patients with IPF. In cohort A, 154 patients were administrated with lebrikizumab or placebo randomly; 351 patients with pirfenidone treatment received lebrikizumab or placebo randomly in cohort B. The mAb showed well-tolerance and safety; however, its administration was not associated with decreased FVC. Both the cohorts did not meet the main endpoints.
Romilkimab (SAR156597)	IL-13 and IL-4	dcSSc	Antibody	The phase II randomized, double-blind, placebo-controlled trial (NCT02921971) reported that the mAb significantly lowered the least-squares mean change in mRSS in dcSSc patients. No cardiac safety signals were shown in romilkimab treatment group. Two death cases in the trial neither were considered treatment related. Another phase IIb trial (NCT02345070) showed that romilkimab did not meet the primary and secondary endpoints in treatment of IPF patients.
Ianalumab (VAY-736)	BAFF-R	IPF	Antibody	A phase II triple-blind, placebo-controlled trial (NCT03287414) is ongoing in IPF patients. The primary endpoint of the trial is the change of FVC rate at 48-week treatment.
Tocilizumab	IL-6R	SSc	Antibody	Although tocilizumab failed to improve skin thickening, it led less decline in FVC in SSc patients in a phase II trial (NCT01532869), indicating that the antibody may inhibit pulmonary fibrosis. In the phase III trial (NCT02453256), tocilizumab showed its ability to preserve lung function with improved FVC rate in patients with elevated acute-phase reactants and early SSc-ILD. Long-term safety of the antibody was also verified.
Adalimumab	TNF-α	IPF	Antibody	The phase II trial (NCT00786201) was conducted to verify the effect of carlumab in IPF patients. No significant changes in SGRQ total score, DLCO, and FVC indicated that it provides no benefit to IPF patients.
Rituximab	CD20	IPF patients with autoantibodies against epithelial cells (HEp-2 cells)	Antibody	Rituximab is used for lymphoma and leukemia treatment. ILD-related lung fibrosis is associated with immune cell overactivation. Pharmacodynamic study of rituximab in 58 patients with IPF (NCT01969409) is ongoing. Only IPF patients with autoantibodies against epithelial cells (HEp-2 cells) are enrolled. They received 2 doses of rituximab intravenously every 2 weeks, with a maximum treatment duration of 9 months. The primary endpoint is the change in HEp-2 antibody titers. The results are currently awaiting.
Simtuzumab	LOXL2	IPF	Antibody	LOXL2 is pivotal in stabilizing the ECM and facilitating the cross-linking of type I collagen molecules, which is essential for the formation of a structured scaffold that supports fibroblast growth and tissue integrity. The antibody did not change the SGRQ total score, DLCO, and FVC and failed to improve progression-free survival of IPF patients in a phase II trial (NCT01769196). It is also ineffective in alleviating bridging fibrosis of patients with NASH (NCT01672879).
Pamrevlumab (FG-3019)	CTGF	IPF	Antibody	In 2016, an open-label phase II trial (NCT01262001) showed that FG-3019 administration by intravenous infusion can ameliorate FVC of IPF patients, which facilitated following studies. The effectiveness of FG-3019 to change FVC rate, HRCT, and SGRQ total score was further verified by the phase II PRAISE trial (NCT01890265). Then, the phase 3 randomized trial ZEPHYRUS-1 (NCT03955146) was conducted in IPF patients who were not administrating with nintedanib or pirfenidone. However, there was no significance of FVC between treatment and control group.
BG00011 (STX-100)	Integrin vβ6	IPF	Antibody	Based on a preliminary trial that evaluated the safety and tolerability of BG00011 (NCT01371305), a phase IIb, randomized, double-blind, placebo-controlled study (NCT03573505) was initiated to assess its safety profile and clinical efficacy in patients with IPF. In this study, BG00011 was administered subcutaneously at a dosage of 56 mg once weekly for a duration of 52 weeks, with the primary outcome measure being the annual rate of change in FVC. However, the study was terminated in September 2019 due to safety concerns.
Rilonacept	IL-1	dcSSc	Fusion protein	The fusion protein is a mimic of IL-1 antibody that blocks the free IL-1. A phase I/II biomarker trial (NCT01538719) was conducted to evaluate the efficiency against skin fibrosis of patients with dcSSc. Unfortunately, no treatment-related effect was observed including the expression of the 2G SSc gene biomarkers and the level of serum IL-6, CRP, and CCL18.
Cenicriviroc (TAK-652)	CCR2/CCR5	Liver fibrosis in adults with NASH	Small molecule (antagonist)	Cenicriviroc has greater effect to slow down the development of liver fibrosis in patients with NASH in the phase 2b CENTAUR Study (NCT02217475). However, the AURORA phase III randomized study (NCT03028740) did not show its efficacy to treat liver fibrosis. Both trials demonstrated the safety and well-tolerance of cenicriviroc.

BAFF, B cell activating factor; BAFF-R, B cell activating factor receptor; CCL, chemokine (C-C motif) ligand; CCR, C-C chemokine receptor; COMP, cartilage oligomeric protein; CRP, C-reactive protein; CTGF, connective tissue growth factor; dcSSc, diffuse cutaneous systemic sclerosis; DLCO, diffusing capacity of the lung for carbon monoxide; FDA, Food and Drug Administration; HRCT, high-resolution computed tomography; ILD, interstitial lung disease; IPF, idiopathic pulmonary fibrosis; LOXL2, lysyl oxidase-like 2; mAb, monoclonal antibody; mRSS, modified Rodnan skin score; NASH, nonalcoholic steatohepatitis; ODD, orphan drug designation; SGRQ, St George’s Respiratory Questionnaire; SLE, systemic lupus erythematosus; SR-FSGS, steroid-resistant focal segmental glomerulosclerosis; SSc, systemic sclerosis; SSc-ILD, systemic sclerosis-associated interstitial lung disease; TGF-β, transforming growth factor-β; THBS1, thrombospondin-1

In addition, the immunotherapies against fibroblasts also show promising effects to inhibit fibrosis. For example, CAR-T cells targeting fibroblast activation protein (FAP) inhibit cardiac fibrosis and restore heart function by eliminating myofibroblasts in a mouse model [163]. FAP CAR mRNA delivery is also a viable alternative, avoiding in vitro culture and adoptive T cell transfer in mice [164]. Computer-assisted epitope prediction has identified a disintegrin and metalloprotease 12 (ADAM12) and GLI1 as key fibroblast markers, presented by MHC class I molecules. Vaccines targeting ADAM12 and GLI1 have shown potential in mobilizing cytotoxic T cells to eliminate fibroblasts and prevent pulmonary fibrosis in animal models, indicating that vaccination may be a potential anti-fibrotic strategy [165].

A comprehensive understanding of fibrosis mechanisms may lead to more effective immunotherapies. Single-cell multi-omics approaches have identified new cell subsets involved in fibrosis progression, such as TREM2^+^CD9^+^ macrophages, segregated nucleus-containing atypical monocytes (SatMs), and ACKR1^+^PLVAP^+^ endothelial cells [166]. In summary, new technologies facilitate novel discovery of unveiled accelerators of fibrosis, which may become new targets for immunotherapies.

### Skin aging

Skin aging, driven by time and environmental factors, manifests as structural, cellular, and molecular changes. It involves reduced cellular proliferation, altered dermal components, and decreased ECM elements, such as collagen, elastin, and glycosaminoglycans. Furthermore, mitochondrial damage and inflammation exacerbate ROS-induced skin damage [167]. Senescent immune cells in the skin reshape the microenvironment, creating a state of low-grade and hyporesponsive immunity. Increased Th17 cells, macrophages, and SASPs worsen inflammation and disrupt skin microbiota balance, while decreased cytotoxic CD4^+^ T cells and NK cells hinder senescent cell clearance [168]. Skin aging has been linked to systemic aging and related diseases in preclinical models. Thus, treating skin aging is not only cosmetically significant but also highly valuable for health [169]. Therefore, the therapies for skin aging are valuable for both cosmetic and systemic health outcomes.

Recent investigations to decipher the key molecules and cells in aging skin provide good targets for immunotherapy. Solá et al. [170] found that aged skin has more IL-17-producing immunocytes than young skin by single-cell RNA sequencing. Anti-IL-17A/F antibodies can inhibit the IL-17A/F-NF-κB pathway to prevent inflammation and delay epidermal aging in aged mice. CD4^+^ cytotoxic T cell subset has been identified as key immune cells for clearing senescent skin cells [171]. These CTLs, drawn by CCL9, patrol the skin and kill senescent cells by recognizing human cytomegalovirus-derived glycoprotein B (HCMV-gB). Adoptive transfer using CD4^+^ CTLs or HCMV-gB-CAR CD4^+^ CTLs in a mouse model is being explored. As exogenous antigens, HCMV-gB accumulates in SCs since HCMV can establish a lifelong latent infection and reactivate during aging. Vaccine against HCMV-gB may elevate the efficiency of senescent cell clearance in elderly with HCMV infection.

### Cancer

**Fig. 5.**
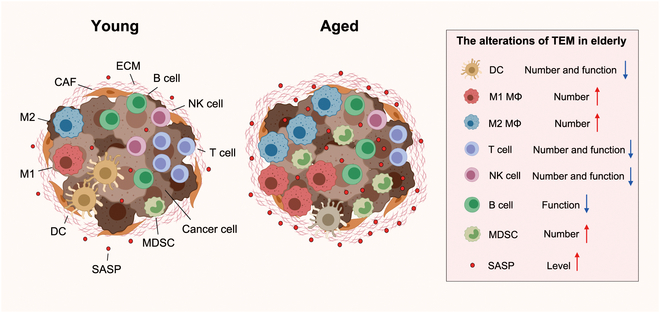
The alterations of tumor microenvironment (TME) in older patients. Immunosenescence leads to changes in the immune system that reshapes TME. The decline of the numbers and functions of antigen-presenting cells (APCs), T cells, and TCR polymorphism abates the antitumor immunity. Myelopoiesis increases give rise to abundance of MDSCs that produce immunosuppressive factors. In addition, the increase of SASPs like IL-6, IL-1, and TNF facilitates cancer progression by activating related signal pathways. All the changes may impact the effectiveness of cancer immunotherapy in elderly. The figure is created with MedPeer (medpeer.cn).

Cancer is closely related to aging. The incidence of cancer continues to rise until the age of 85, highlighting the crucial role of aging in cancer development [172]. Aging promotes cancer through mechanisms such as oxidative stress, accumulation of DNA damage, increased senescent cells and SASPs, immunosenescence, stem cell exhaustion and dysfunction, genomic instability, epigenetic changes, and ECM remodeling. Population aging will lead to a rapid increase in cancer incidence. The decline in immune function in elderly patients and age-related immune dysfunction create a pro-tumor microenvironment, thereby reducing the efficacy of immunotherapy in elderly cancer patients (see Fig. 5). Age-related immune dysfunction includes a reduction in DCs and TCR repertoire, an increase in M2 macrophages and MDSCs, up-regulation of inflammatory cytokine levels, and changes in the ECM, all of which may affect the efficacy of cancer immunotherapy. Therefore, remodeling the tumor microenvironment is a key approach to enhancing antitumor immunity in elderly cancer patients.

The great success of ICB in cancer treatment highlights the importance of tumor microenvironment remodeling. Immune checkpoint inhibitors can relieve the immunosuppression of tumor cells on T cells, enhance the immune system’s attack on tumor cells, and also help clear senescent cells. Nivolumab (PD-1 mAb), ipilimumab (anti- CTLA-4 mAb), and relatlimab (anti-LAG-3 mAb) have been proven effective in controlling tumor growth in multiple clinical trials. In ICB therapy, clinical evidence reveals a paradoxical phenomenon: Elderly cancer patients often show higher sensitivity. A meta-analysis of 25 ICB trials indicated that elderly patients had a better response rate than younger patients [173]. This is attributed to an Akkermansia-centered gut microbiome configuration (enterotype E) unique to older individuals, which enhances the dendritic cell–CD8^+^ T cell axis and thereby provides a novel microbiome-based target for precision immunotherapy. Despite this promising phenomenon in clinical experiments of ICB therapy, preclinical models have revealed limitations. In elderly mice, PD-1/CTLA-4 inhibitors failed to eradicate tumors, which was associated with a decline in DC activation capacity and CD8^+^ T cell exhaustion [174]. This discrepancy between clinical and preclinical results highlights the complexity of translating age-specific mechanisms into practical applications.

The accumulation of age-related immunosuppressive TREM2^+^ macrophages is associated with ICB resistance, as observed in a metastatic cancer cohort [175]. Targeting these cells with cell cycle-dependent kinase inhibitors or nanoregulators that can disrupt mitochondrial calcium homeostasis may reverse immunosuppression. Additionally, inhibiting IL-1R signaling with antibodies can disrupt the generation of IL-1α-producing monocyte-derived macrophages and prolong the overall survival of elderly cancer patients [176]. During aging, the number and function of bone marrow HSC change, leading to increased myeloid output and elevated MDSC numbers [177]. MDSCs are important immunosuppressive cells in the tumor microenvironment. They suppress the function and proliferation of T cells by secreting immunosuppressive factors (such as IL-10 and TGF-β), consuming l-arginine, and generating ROS, thereby weakening the antitumor immune response [178]. Therapies targeting myeloid cells have been shown to improve tumor control and the cytotoxicity of CD8^+^ T lymphocytes in elderly patients by activating conventional type 1 DC (cDC1), indicating the potential of rejuvenating senescent immune cells [179]. In recent years, with the deepening understanding of the relationship between senescent cells and immunotherapy, as well as the hindrance of senescent cells and SASPs to tumor immunity, anti-aging therapies have emerged as a new anti-cancer treatment approach. Many studies have confirmed that eliminating senescent cells and SASP can improve the tumor microenvironment and enhance antitumor immune responses [180]. A study developed a nanotechnology-based platform (mGL392) that precisely targets lipofuscin in senescent cells for clearance. Experiments demonstrated that mGL392 could significantly reduce the accumulation of senescent cells, lower the risk of recurrence, and exhibit excellent anti-cancer effects in a melanoma model [181]. Additionally, blocking senescence-related factors such as BCL-2 and mTOR can inhibit tumor growth and neutralize drug resistance [182,183]. Eliminating SASP (such as anti-IL-6 antibodies) can synergize with immune checkpoint inhibitors to promote antitumor immunity and eliminate irAEs, which may be applicable to elderly patients resistant to immunotherapy [184]. Moreover, conventional therapies (e.g., radiation and chemotherapy) often drive tumor cells into senescence. Recent work positions therapy-induced senescence followed by selective senescent cell removal as an adjunct to standard cancer therapy, enhancing efficacy, reducing relapse, and limiting side effects [185]. While senolytic or senescence-modulating agents, including navitoclax, have shown tumor suppression in animal models, they carry acute toxicities—most notably dose-limiting thrombocytopenia and neutropenia [185]. Combining senescent cell-clearing immunotherapies with standard oncologic treatments offers a promising strategy to mitigate these liabilities.

Despite the advancement of cancer immunotherapy for elderly, related clinical translation faces hurdles, including underrepresentation of elderly patients in clinical trials. An analysis of FDA-registered ICB trials (2018–2022) revealed inadequate inclusion of older adults, particularly those with comorbidities or frailty [186]. This gap limits data on dose optimization, toxicity management, and biomarkers. For instance, neoantigen burden—a predictor of ICB response—may decline with age due to reduced DNA repair efficiency, yet quantification of immunoediting patterns suggests that age-specific neoantigen landscapes could serve as predictive biomarkers [187].

In conclusion, optimizing immunotherapy for elderly patients requires a dual focus: (a) deciphering age-specific interactions between immunosenescence, the tumor microenvironment, and treatment modalities, and (b) designing clinical trials with robust geriatric assessments. Leveraging nanotechnology, senescence biology, and precision biomarkers will be critical to unlocking the full potential of cancer immunotherapy for elderly patients.

## Discussion and Perspectives

The biochemical aging program typically begins when molecular-level primary damage triggers cellular senescence; once age-related immune surveillance wanes, SASP-driven inflammation amplifies the damage, promotes fibrosis, exhausts organ reserve, and culminates in age-related diseases. Compared with chemicals regulating age-related pathways, immunotherapy can precisely eliminate senescent cells and reshape the immune microenvironment. Senescent cells overexpress proteins like CD153, GPNMB, IL-11, uPAR, and PD-L1, which can be targeted by CAR-T cells, antibodies, and vaccines for selective elimination [8,14,18,19,23]. Methods such as NK cell injection, my-HSC depletion, and inflammatory cytokine blockade have shown promise for rejuvenating the immune system in animal models and clinical trials [28,39]. However, immunotherapies for age-related diseases must consider their unique pathologies and select appropriate targets. Despite the potential of SC clearance and immune reshaping, immunotherapies face challenges, including the following: (a) Heterogeneous senescent cell and SASP targets in different elderly populations make it hard to find universal aging targets, leading to therapy ineffectiveness in some individuals. (b) Immunosenescence affects the efficiency of immunotherapy by attenuated immune function and adoptive cell exhaustion (Table 5). Although immune reprogramming is possible, full immune restoration remains challenging. (c) Specific immunotherapies may harm nonsenescent cells expressing age-related molecules. As most aging markers are senescence-associated rather than specific antigens, such therapies can cause severe side effects by eliminating nonsenescent cells. Identifying aging-specific antigens is key to solving this problem. (d) Senescent cell and SASP clearance might disrupt physiological processes and even accelerate aging, as some senescent cells and SASPs are vital for tissue function. (e) Immunotherapy issues may be more severe in elderly patients. Vaccines with low immunogenicity and MHC affinity may fail due to immunosenescence. Moreover, CAR-T therapy can cause fatal cytokine storms in those with weakened compensatory and repair abilities. Similar obstacles exist for age-related disease immunotherapies, which also have more complex etiologies. Further research is essential to understand aging-driven disease development and lay the groundwork for prevention and treatment.

**Table 5.** Mechanisms of immunosenescence affecting the efficiency of immunotherapy

Defect type	Manifestation	Effects on immunotherapy
The shrinking of T lymphocyte repertoire	Thymus involution; decrease of CD8^+^ T cells; the loss of surface CD28 expression; increase of memory T cells	Impaired neoantigen recognition and T cell expansion lead to weak vaccine-induced CTL responses
B cell dysfunction	Myeloid-biased differentiation of HSCs; reduced mature B cells with impaired antibody class switching	Low antibody titers and poor affinity after vaccination
Impaired antigen presentation	Reduced DC numbers; attenuated TLR signaling; decreased costimulatory molecule expression	Decreased efficiency of vaccine antigen uptake and presentation; insufficient activation after ACT
Impaired neutrophil chemotaxis	Significantly reduced directional accuracy of neutrophil migration	Insufficient immune cell recruitment at the vaccination site
The imbalance of macrophage polarization	Impaired M1/M2 switching with a persistent pro-inflammatory state	Influence the status of immunocytes from ACT or induced by vaccines
Attenuated cytotoxicity	Decreased NK cell and T cell cytotoxicity	Compromises the efficiency of CAR-T or NK cell-mediated clearance of senescent or tumor cells in adoptive transfer of autologous lymphocytes
Inflammaging	Elevated SASP levels and PD-1 expression	Impaired differentiation of B cells into plasma cells, resulting in reduced antibody production; induction of T cell exhaustion
Metabolic reprogramming	Reduced glycolytic in T cells; restricted T cell proliferation and function	Attenuated T cell response by vaccination; adoptive cell reprogramming

Beyond senescent cells, SASPs, and other characteristic aging molecules, longevity-associated genes play crucial roles in counteracting aging across multiple physiological systems, particularly within the immune, vascular, and cardiac systems. This indicates significant promise for immunotherapies targeting these genes to induce rejuvenation. Specific longevity-associated genes, such as Sirtuins, RGP1, CASP8, and DIMT1, reduce cellular damage and stress by regulating oxidative stress responses, DNA repair, and protein homeostasis [188,189]. LAV-BPIFB4, prevalent in long-lived individuals, improves microvascular structure and function, restores pericyte function, and effectively slows cardiovascular aging [190]. Furthermore, mitochondrial translation genes, along with genes involved in proteolysis and PI3K–Akt signaling, have been demonstrated to contribute to an extended lifespan [188,191]. Epigenetically, regulators like SET-26, HCF-1, and FLOT1 modulate DNA replication, metabolism, and inflammation through chromatin remodeling and epigenetic modifications, affecting lifespan and the rate of epigenetic aging acceleration [192,193]. To date, no immunotherapy targeting longevity-associated genes has entered clinical trials for aging. Future immunotherapies may therefore integrate strategies such as reprogramming-induced rejuvenation or fecal microbiota transplantation (FMT) targeting the gut microbiome to restore systemic immune competence and combat vascular and cardiac aging [194].

Multi-omics analysis of aging is crucial for future research, diagnosis, and treatment. For example, Ma et al. [195] created a high-resolution spatial transcriptomic atlas of aging, revealing that increased tissue structural entropyand loss of integrity drive the formation of aging-sensitive spots, characterized by IgG-secreting cell accumulation. This offers new tissue-level aging intervention strategies. Another team’s protein atlas of aging highlighted changes in many ECM and mitochondrial membrane proteins.

According to the Information Theory of Aging, aging can be reversed by epigenetic reprogramming, with epigenomics helping to uncover environmental exposure effects on aging [196]. All multi-omics data can be integrated into a central “senomics” platform for discovering new targets for aging intervention.

**Fig. 6.**
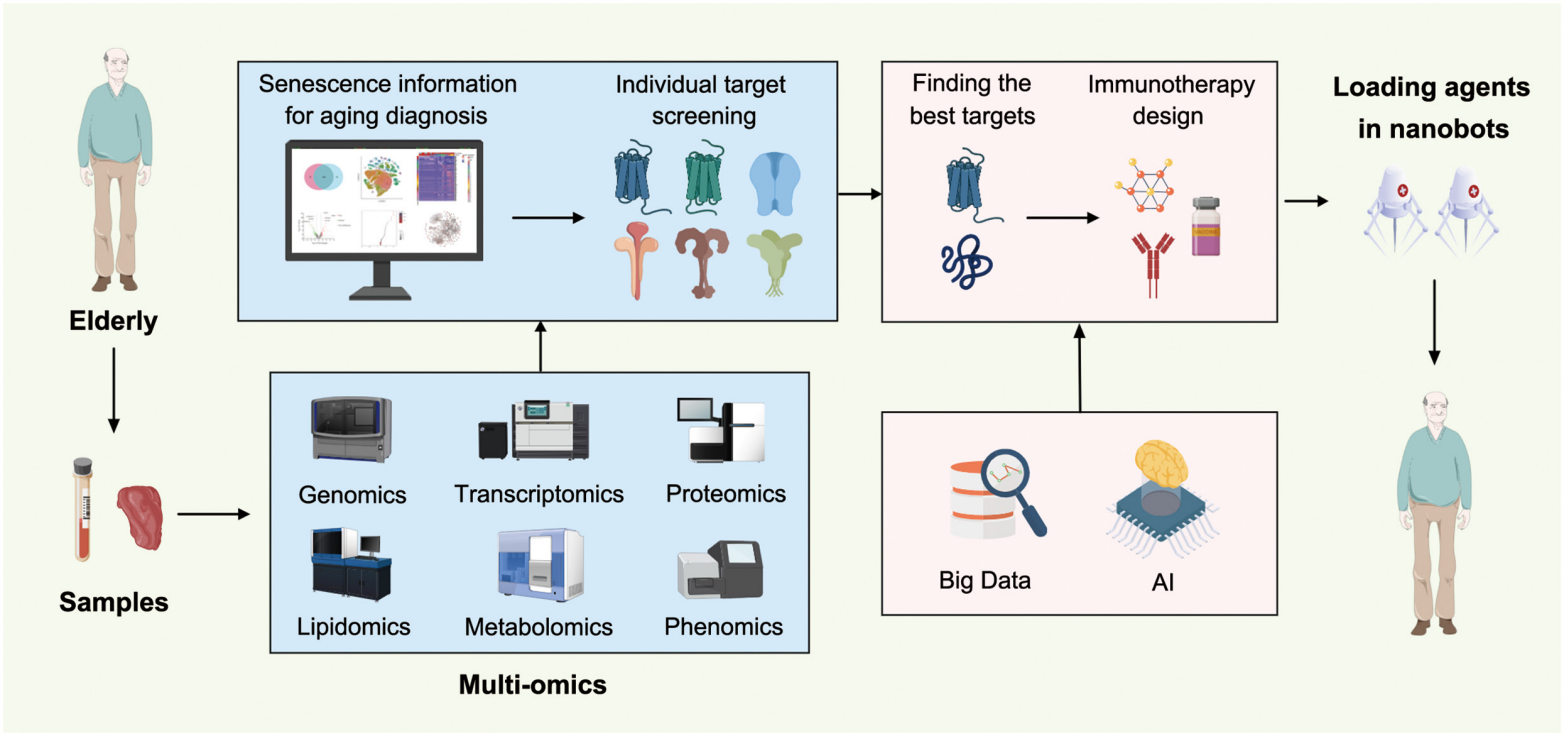
The process of individual aging treatments in the future. The samples of elderly can be measured and analyzed via multi-omics, which supplies the information of senescence for aging diagnosis and sets foundation of the screening of individual aging targets. Then, Big Data and AI can help us find the best targets and design corresponding immunotherapy agents. At last, the selected agents are carried by nanobots and administrated into elderly for aging treatment and real-time detection of senescence omics spectrum. The figure is created with MedPeer (medpeer.cn).

With technological advances, personalized treatment platforms are the future of aging therapy, helping to overcome the current hurdles (Fig. 6). First, there’s an urgent need for diagnostic criteria for aging-related biomarkers. Despite much research, in vivo senescent cell identification and characterization face conceptual, methodological, and practical challenges. To address this, some studies have proposed minimal information for in vivo senescence experiments (MIIVSE), which leverages multi-omics and advanced analytical tools to better assess in vivo cellular senescence biomarkers. The classical senescence markers initially identified and established in cell culture models have limited utility in natural tissue microenvironments or “in situ” experiments. Therefore, the minimum information for cellular senescence experimentation in vivo (MICSE) is proposed to improve the evaluation of cellular senescence biomarkers in vivo by multi-omics (containing genomics, transcriptomics, proteomics, lipidomics, metabolomics, and phenomics) and advanced analysis tools (including high content analysis, methods of multi-omics data integration, and machine learning) [197]. Using MIIVSE and MICSE data, AI can detect and assess patients’ aging status. Individual senomic maps can then be used to find suitable immunotherapy targets by evaluating factors like expression abundance, MHC affinity, immunogenicity, cross-reactivity, and physiological function. Next, AI can assist to design drugs targeting aging-related molecules and predict next-generation antibodies and antigens using computational protein design and machine learning [198]. Finally, nanorobots are being developed to monitor senomic profiles in real time and deliver drugs to aging-affected areas for immunotherapy.

Because immune responses are recognized to differ markedly between sexes, all immunotherapeutic strategies for aging and age-related diseases should consider sex stratification where feasible. Women typically display more robust innate and adaptive immunity and higher vaccine-induced antibody titers, but are more susceptible to autoimmune disorders; men, in contrast, mount weaker inflammatory responses, recover more slowly from infection, and are at greater risk of severe infection-related complications [199].

In conclusion, immunotherapy for aging and age-related diseases faces many challenges. Fully understanding the link between aging and age-related diseases is vital for developing new treatments. Personalized immunotherapy, based on senomics, bioinformatics, AI, and nanotechnology, may transform aging treatment and promote healthy aging.
